# Fisetin induces DNA double-strand break and interferes with the repair of radiation-induced damage to radiosensitize triple negative breast cancer cells

**DOI:** 10.1186/s13046-022-02442-x

**Published:** 2022-08-22

**Authors:** Shayan Khozooei, Konstanze Lettau, Francesca Barletta, Tina Jost, Simone Rebholz, Soundaram Veerappan, Mirita Franz-Wachtel, Boris Macek, George Iliakis, Luitpold V. Distel, Daniel Zips, Mahmoud Toulany

**Affiliations:** 1 Division of Radiobiology and Molecular Environmental Research, Department of Radiation Oncology, University of Tuebingen, Roentgenweg 11, 72076 Tuebingen, Germany; 2 German Cancer Consortium (DKTK), partner site Tuebingen, German Cancer Research Center (DKFZ), Heidelberg, Germany; 3 Quantitative Biology Center (QBiC), University of Tuebingen, Auf der Morgenstelle 10, 72076 Tuebingen, Germany; 4 Department of Radiation Oncology, Universitätsklinikum Erlangen, Friedrich-Alexander-Universität Erlangen-Nürnberg, Erlangen, Germany; 5 Comprehensive Cancer Center Erlangen-EMN (CCC ER-EMN), Universitätsklinikum Erlangen, Friedrich-Alexander-Universität Erlangen-Nürnberg, Erlangen, Germany; 6 Proteome Center Tuebingen, University of Tuebingen, Tuebingen, Germany; 7 Divison of Experimental Radiation Oncology, Department of Radiation Oncology, Institutsgruppe 1, Bauteil A, 4.038, University of Duisburg-Essen, Hufelandstr. 55, 45122 Essen, Germany

**Keywords:** Triple negative breast cancer, Y-box binding protein-1, Fisetin, Double strand break repair, Radiosensitization

## Abstract

**Background:**

Triple-negative breast cancer (TNBC) is associated with aggressiveness and a poor prognosis. Besides surgery, radiotherapy serves as the major treatment modality for TNBC. However, response to radiotherapy is limited in many patients, most likely because of DNA damage response (DDR) signaling mediated radioresistance. Y-box binding protein-1 (YB-1) is a multifunctional protein that regulates the cancer hallmarks among them resisting to radiotherapy-induced cell death. Fisetin, is a plant flavonol of the flavonoid family of plant polyphenols that has anticancer properties, partially through inhibition of p90 ribosomal S6 kinase (RSK)-mediated YB-1 phosphorylation. The combination of fisetin with radiotherapy has not yet been investigated.

**Methods:**

Activation status of the RSK signaling pathway in total cell lysate and in the subcellular fractions was analyzed by Western blotting. Standard clonogenic assay was applied to test post-irradiation cell survival. γH2AX foci assay and 3 color fluorescence in situ hybridization analyses were performed to study frequency of double-strand breaks (DSB) and chromosomal aberrations, respectively. The underlying repair pathways targeted by fisetin were studied in cells expressing genomically integrated reporter constructs for the DSB repair pathways via quantifying the expression of green fluorescence protein by flow cytometry. Flow cytometric quantification of sub-G1 cells and the protein expression of LC3-II were employed to measure apoptosis and autophagy, respectively. Kinase array and phosphoproteomics were performed to study the effect of fisetin on DDR response signaling.

**Results:**

We showed that the effect of fisetin on YB-1 phosphorylation in TNBC cells is comparable to the effect of the RSK pharmacological inhibitors. Similar to ionizing radiation (IR), fisetin induces DSB. Additionally, fisetin impairs repair of IR-induced DSB through suppressing the classical non-homologous end-joining and homologous recombination repair pathways, leading to chromosomal aberration as tested by metaphase analysis. Effect of fisetin on DSB repair was partially dependent on YB-1 expression. Phosphoproteomic analysis revealed that fisetin inhibits DDR signaling*,* which leads to radiosensitization in TNBC cells, as shown in combination with single dose or fractionated doses irradiation.

**Conclusion:**

Fisetin acts as a DSB-inducing agent and simultaneously inhibits repair of IR-induced DSB. Thus, fisetin may serve as an effective therapeutic strategy to improve TNBC radiotherapy outcome.

**Supplementary Information:**

The online version contains supplementary material available at 10.1186/s13046-022-02442-x.

## Background

Triple negative breast cancer (TNBC) does not express estrogen receptor (ER) and progesterone receptor (PR) and is characterized by the absence of HER2 overexpression/ amplification [1]. TNBC is one of the most aggressive subtypes of breast cancer that accounts for about 20% of breast cancers. Since the three receptors are the major target of most hormone therapies, treating patients with TNBC remains challenging. Radiotherapy, as an important treatment approach for breast cancer patients, improves locoregional control both after breast conserving surgery and mastectomy [2], with a positive impact in high-risk patients for long-term survival [1]. Tumor radioresistance comprised of acquired radioresistance as well as intrinsic radioresistance is the major cause of a diminished radiotherapy outcome.

Y-box binding protein 1 (YB-1), is a member of the cold-shock protein superfamily. The protein contains a cold-shock domain (CSD) that enables it to bind to DNA and RNA [3]. YB-1 is overexpressed in different tumor types and is involved in nearly all cancer hallmarks described to date [4], particularly cell death resistance after exposure to ionizing radiation (IR) [5, 6]. In breast cancers, expression of YB-1 plays an important role in cancer progression from the early-stage; this identifies YB-1 as a potential target for breast cancer treatment [7]. Clinical studies revealed that YB-1 expression diminishes response to radiochemotherapy in different tumor entities [8–10], is crucial in acquired drug resistance development [11] and is associated with tumor recurrence [12]. DNA double-strand break (DSB) is the major cause of radiation-induced cell death. YB-1 knockdown interferes with DSB repair and mediates radiosensitization [5, 6]. In support of the role of YB-1 in DNA repair, YB-1 was found in a complex with MSH2 and Ku80 as well as with WRN proteins, involved in mismatch repair and DSB repair, respectively [13]. In line with the proposed role of YB-1 in DNA repair, namely DSB repair, previous reports have shown that in YB-1 knockdown breast cancer cells, the frequency of residual DSB is increased and the cells become radiosensitized [5, 6]. Because YB-1 lacks a kinase domain, direct molecular targeting by applying pharmacological inhibitors is not plausible. Thus, investigations have focused on targeting p90 ribosomal S6 kinase (RSK) as the most important kinase stimulating YB-1 phosphorylation [14] to interfere with its prosurvival effect. Recently, we demonstrated that in breast cancer cells application of the RSK inhibitor LJI308 effectively blocks YB-1 phosphorylation in non-irradiated as well as in irradiated cells [6]. However, compared to the YB-1-siRNA approach, the effect of LJI308 on inhibition of DSB repair was minimal and it did not induce radiosensitization although YB-1 activity was blocked [6]. Mechanistically, we demonstrated that activation of AKT after RSK inhibition or constitutive activation of AKT in cells with mutation in genes such as *PIK3CA* or *PTEN* stimulates DSB repair and leads to the failure of RSK inhibitors to induce radiosensitization. Supporting these results, we were able to show that the dual inhibition of AKT and RSK is able to induce sensitivity to IR in breast cancer cells independent of TNBC status [6]. The toxicity issue of this approach remains to be investigated in further in vivo studies.

Although successful targeting of YB-1 by other approaches, *i.e.*, RNAi approaches and blocking peptides has been reported, the applicability of these approaches in vivo remains a major issue. Recently, the effect of the plant flavonoid fisetin has been investigated on the activation of YB-1 in tumor cells from different entities [15]. It has been shown that fisetin interferes with binding of RSK2 to YB-1 and that it thus blocks YB-1 phosphorylation [16]. According to the described role of S102 phosphorylated YB-1 in DSB repair [6], fisetin in combination with IR might improve radiation response of TNBC. YB-1 independent targets of fisetin*, e.g.,* demethylating histone H3K36 ﻿[17], inhibition of AKT [18] and modulating autophagy [19] may also affect radiation response, independently of its effect on YB-1. In the present study, the effect of fisetin on phosphorylation of proteins inside and outside the YB-1 cascade was analyzed in TNBC cells. YB-1-dependent and YB-1-independent effect of fisetin in DSB repair were investigated. The obtained data demonstrated that fisetin induces DSB and has a strong anti-clonogenic activity in TNBC cells when applied as monotherapy. Likewise, fisetin strongly blocked DSB repair after irradiation and improved radiosensitivity in a combined therapy.

## Materials and methods

### Cell lines

TNBC cell lines; MDA-MB-231 (ATCC® HTB-26™), MDA-MB-468 (ATCC® HTB-132™), MDA-MB-453 (ATCC® HTB-131™) and HS 578T (ATCC® HTB-126™) as well as non-TNBC cell lines MCF-7 and T47D were used. Single nucleotide polymorphism (SNP) profiling was used to verify the authenticity of the cells (Multiplexion, Heidelberg, Germany). Normal human skin fibroblasts (HSF-7 cells) were included in the study as healthy control cells. The cells, except MCF-7, were cultured in DMEM containing 10% fetal calf serum (FCS) and 1% penicillin–streptomycin (PS) and incubated in a humidified atmosphere of 93% air and 7% CO2 at 37 °C. MCF-7 cells were culture in RPMI medium containing 10% FCS and 1% PS. U2OS osteosarcoma cells expressing genomically integrated reporter constructs for homologous recombination (HR), classical non-homologous end joining (C-NHEJ) and alternative NHEJ (Alt-NHEJ) repair pathways, engineered in Dr. Jeremy Stark's lab [20] were cultured in DMEM containing 10% FCS, 1% PS and 2 µg/ml of puromycin.

### Antibodies and reagents

Primary antibodies against YB-1 (#42,042), phospho-YB-1 (S102) (#2900), phospho-RSK (T359/S363) (#9344), RSK1/RSK2/RSK3 (#9355), phospho-AKT (S473) (#9271) and p62 (#8025) were purchased from Cell Signaling Technology (Frankfurt, Germany). All these antibodies were used at the dilution of 1:1000. LC3 antibody was purchased from Nanotools (Teningen, Germany, dilution 1:150). The β-actin antibody (#A2066, dilution 1:1000) and Triamzinolonacetonid (#8056) were purchased from Sigma-Aldrich (Taufkirchen, Germany). The anti-phospho-H2AX antibody (S139) (#05–636, dilution 1:300) was purchased from Merck (Darmstadt, Germany). The RSK inhibitor LJI308 (#S7871) and fisetin (#S2298) were purchased from Selleckchem (#S7871) (Munich, Germany). BI-D1870 (#BML-EI407) was purchased from Enzo (Lörrach, Germany). Rad51 inhibitor B02 (#S8434) was purchased from Absource (Munich, Germany). Small interfering RNA (siRNA) against YB-1 (#M-010213) and nontargeting siRNA (#D-001810) were purchased from Darmacon (Frankfurt, Germany). Lipofectamine RNAiMAX transfection reagent and opti-MEM were purchased from Invitrogen (Darmstadt, Germany). I-SceI-GR-RFP expression plasmid was a gift from Tom Misteli (Addgene plasmid #17,654, USA).

### Inhibitor treatment

The RSK inhibitors LJI308 (LJI) and BI-D1870 (BID) were dissolved in dimethyl sulfoxide (DMSO). For treatment, stock solutions of the inhibitors were diluted in culture medium and applied to the cells. Control cells received equivalent DMSO concentrations.

### Irradiation

Irradiation was performed at 37 °C using a Gulmay RS225 X-ray machine (Gulmay Limited, Chertsey, UK) at a dose rate of 1 Gy/min operated at 200 kVp, 15 mA and 0.5 mm copper filter.

### Cellular fractionation and immunoblotting analysis

Cytoplasmic and nuclear protein fractions were separated as previously described [21]. Cells were harvested in lysis buffer as described previously [22]. Protein was quantified with Biorad DC™ Protein Assay Reagent and 100 µg of protein were loaded to SDS-PAGE. Afterwards, proteins were transferred to a nitrocellulose membrane, incubated with the corresponding primary antibodies at 4 °C overnight, followed by 3 washes and then incubated with the corresponding secondary antibodies for 1 h at room temperature. PVDF (polyvinylidene fluoride) was used to detect LC3 I/II proteins. LI-COR Biosciences system (Bad Homburg, Germany) and ECL detection kits (GE Healthcare or Cell Signaling) were used to detect chemiluminescence.

### Clonogenic assay

Clonogenic assay was performed to investigate potential radiosensitizing effect of fisetin in all TNBC and HSF-7 cells. Briefly, log phase cells in T12.5 flasks were treated with DMSO (0.1%) control (10 flasks) or 75 µM of Fisetin (10 flasks) for 24 h. Thereafter, cells were clustered in 4 groups, *i.e.*, control, IR, fisetin and fisetin + IR, with 5 flasks/group. Cells were either mock irradiated (control and fisetin groups) or irradiated with one fraction of 1 Gy (IR and IR + fisetin groups). From each group, one flask was trypsinized immediately after irradiation or mock irradiation and plated in 6-well plates in medium containing 20% FCS without additional treatment. The medium was changed for the rest of the cells (16 flasks), with the fresh medium containing DMSO (0.1%) or fisetin for the next fraction of irradiation the following day. The same procedure was repeated on day 2 and the following days. In the experiments with single dose irradiation, cells were treated either with DMSO (0.07%) or fisetin (75 µM). Twenty-four hours later, cells were mock irradiated or irradiated with 0 to 4 Gy. Cells were trypsinized immediately after irradiation or mock irradiation and plated in 6-well plates. Depending on the cell lines, 10 to 15 days later, cultures were stained and colonies with more than 50 cells were scored as survivors. Plating efficiency (PE) in each condition was calculated by dividing number of colonies to the number of seeded cells. The survival fraction for each radiation dose was calculated by dividing the PE of irradiated cells with the PE of non-irradiated DMSO control or non-irradiated fisetin control. Survival curves were graphed based on the calculated survival fractions by using Sigma Plot and Microsoft Excel software.

### γH2AX assay

To determine residual DNA DSB after IR, TNBC and HSF-7 cell lines were irradiated with the indicated dose of X-ray in each experiment. Either thirty minutes or 24 h after irradiation, cells were fixed with 70% ethanol and followed by staining with phospho-H2AX (S139) antibody as described before [23]. The foci were counted using FoCo software [24] and the average foci number per nuclei were determined and graphed.

### SiRNA transfection

SiRNA transfection was performed as described previously [5, ]. Cells were transfected with 50 nM of non-target siRNA or YB-1 siRNA. Twenty-four hours after transfection, cells were treated according to the required experimental procedure.

### Three-color fluorescence in situ hybridization

The effect of fisetin on chromosomal aberration was studied with three-color fluorescence in situ hybridization as described before [25] in MDA-MB-231 and MDA-MB-468 cells with differential effect of fisetin on YB-1 phosphorylation. Cells were seeded in T175 culture flasks and 24 h later were treated with DMSO (0.07%) or fisetin (75 µM) for  72 h hours. In the IR condition, the cells were irradiated with 2 Gy and after 48 h, a mitotic shake off was performed to detach the currently dividing cells with condensed chromosomes mitotic cells. After cell lysis, pellets were resuspended in 0.02% potassium chloride solution (Sigma Aldrich, München, Germany) and a 3:1 methanol:acetic acid (Sigma Aldrich, München, Germany) solution. DNA was transferred to glass slides and treated with RNase (Roche, Penzberg, Germany) and pepsin (Sigma Aldrich, München, Germany) to remove cell debris. Afterwards, DNA was fixated with formaldehyde-buffer (Merck, Darmstadt, Germany), denatured with formamide-buffer (Merck, Darmstadt, Germany) at 72 °C and hybridization was performed by incubating DNA with a mixture of probes for chromosome #1, #2 and #4 at 37 °C for 72 h. Finally, glass slides were stained with FITC (Merck, Darmstadt, Germany) and anti-avidin/rhodamin (Merck, Darmstadt, Germany) and microscopic images were taken using a Zeiss Axioplan 2 fluorescence microscope. The Metasystems software (Metafer 4 V3.10.1, Altlussheim, Germany) was used to search chromosome metaphases automatically at 100 × magnification and an image of each metaphase was acquired at a magnification of 630 × . For each metaphase, black and white images of each color (red, green and blue) were acquired and used for evaluation.

### DSB repair pathway analysis

The DSB repair pathway analysis was performed in U2OS cells expressing genomically integrated reporter constructs for HR, C-NHEJ and Alt-NHEJ repair pathways [20]. Cells (5 × 10^5^) were transfected with 800 ng/ml of inducible I-SceI expression plasmid [26] and were treated with fisetin (75 µM) after 24 h. Twenty-four hours after the fisetin treatment, the cells were treated with 100 ng/ml of triamcinolonacetonid (TA) to induce nuclear translocation of I-SceI. After an additional 24 h, cells were analyzed for GFP expression by BD FACSCanto™ System using Flowing software.

### Flow cytometric analysis of apoptosis

To analyze the effect of fisetin on apoptosis and cell cycle progression, all TNBC cell lines were seeded 24 h before treatment with DMSO (0.07%) or fisetin (75 µM). After twenty-four hours, cultures were mock irradiated or irradiated with 4 Gy. Cells were trypsinized and fixed with 70% ethanol 48 h after irradiation. Propidium iodide staining and cell preparation for cell cycle analysis were performed as described before [27] using BD FACSCanto™ System and the data were analyzed by FlowJo software.

### Phospho-kinase proteome profiler array

MDA-MB-231 cells were treated with DMSO (0.07%) or fisetin (75 µM) for 72 h and followed by mock irradiation or irradiation with 4 Gy. Protein samples were extracted at 30 min and 24 h after irradiation. Phospho-kinase proteome array was performed according to the manufacturer’s protocol (R&D Systems, Minneapolis, MN, USA).

### SILAC-based phosphoproteomics analysis

Quantitative phosphoproteomics using Stable Isotope Labeling by Amino Acids in Cell Culture (SILAC) was done in 6 samples distributed in two experimental groups in biological triplicates for MDA-MB-468 as follow: 1) IR (4 Gy) (“light” and “medium” labelled); 2) IR plus Fisetin (75 µM) (IR + Fisetin) (“heavy labelled). Similar treatments were performed in MDA-MB-231 with the exception of labelling IR condition with “light” medium. Briefly, MDA-MB-231 and MDA-MB-468 cell lines were grown in 3 different media containing”light” (Lys0, Arg0), “medium-heavy” (^13^C_6_^14^N_4_-L-arginine/Arg6, 4,4,5,5-D4-L-lysine/Lys4) and “heavy” (^13^C_6_^15^N_4_-L arginine/Arg10,^13^C_6_^15^N_2_-L-Lysine/Lys8) amino acids. Cells were cultured for 10 passages to ensure the incorporation of labeled amino acids was higher than 97% in all cases. Afterwards, IR treatment was applied for 30 min and cells were lysed in lysis buffer [28] for 30 min at room temperature and then sonified for 1 min on ice (Bandelin SONOPULS HD 200, Program MS73D). Protein extracts were precipitated with ice-cold acetone-methanol at -20 °C overnight. The proteins were pelleted by centrifugation (2000 × *g*, 20 min, 4 °C) and washed three times with 80% ice-cold acetone. Dried proteins were resolved in digestion buffer (6 M urea, 2 M thiourea, 10 mM Tris, pH 8.0). The samples were mixed in 1:1:1 ratio according to measured protein amounts in two pools, each of them containing a “light- “, “heavy-medium- “ and “heavy-SILAC” sample. Afterwards, 600 µg of the mixture was digested in solution with trypsin as described previously [29] and 3% of the resulting peptides were directly desalted with C_18_ StageTips [30].

The rest of the peptide mixture was purified on Sep-Pak 18 cartridges (Waters) and subjected to phosphopeptide enrichment by MagReSyn Ti-IMAC (ReSyn Bioscience) as described previously [29] with minor modifications: approximately 60 μl of magnetic bead suspension per mix and enrichment round was washed two times for 5 min with 70% ethanol, followed by washing for 10 min with 1% NH_4_OH. Before peptide loading, beads were equilibrated three times with loading buffer (1 M glycolic acid and 5% TFA in 80% ACN). Elution from the beads was performed three times with 100 µl of 1% NH_4_OH. The pooled eluates were further purified by C_18_ StageTips. Peptide mixes were subjected to three consecutive rounds of enrichment. LC–MS analyses of peptides and enriched phosphopeptides were performed on an EASY-nLC 1200 UHPLC coupled to a quadrupole Orbitrap Exploris 480 mass spectrometer (both Thermo Scientific).

Separations of the peptide mixtures and enriched phosphopeptides were performed as described previously [31] with slight modifications: the peptides were injected onto the column in HPLC solvent A (0.1% formic acid) at a flow rate of 500 nl/min and subsequently eluted with a 127 or 57 min segmented gradient of 10–33-50–90% of HPLC solvent B (80% acetonitrile in 0.1% formic acid) at a flow rate of 200 nl/min. The mass spectrometer was operated in data‐dependent mode, collecting MS spectra in the Orbitrap mass analyzer (60,000 resolution, 300–1750 m*/z* range) with an automatic gain control (AGC) set to standard and a maximum ion injection time set to automatic. The 20 most intense precursor ions were sequentially fragmented with a normalized collision energy of 28 in each scan cycle using higher energy collisional dissociation (HCD) fragmentation. In all proteome and phosphoproteome measurements, sequenced precursor masses were excluded from further selection for 30 s. MS/MS spectra were recorded with a resolution of 15,000 and 30,000, respectively, whereby AGC was set to standard and fill time was set to automatic.

MS data were processed using default parameters of the MaxQuant software (v1.5.2.8) [32]. Extracted peak lists were submitted to a database search using the Andromeda search engine [33] to query a target-decoy [34] database of *homo sapiens* (97,795 entries, downloaded on 7th of October 2020) and 285 commonly observed contaminants.

In the database search, full tryptic specificity was required and up to two missed cleavages were allowed. Protein N-terminal acetylation, oxidation of methionine, and phosphorylation of serine, threonine, and tyrosine were set as variable modifications, whereas no fixed modification was defined. Initial precursor mass tolerance was set to 4.5 ppm, and 20 ppm at the fragment ion level. Peptide, protein and modification site identifications were filtered at a false discovery rate (FDR) of 0.01. For protein group quantitation a minimum of two quantified non-phosphorylated peptides were required, for phosphorylation sites at least one quantitation event was required. Quantified phosphorylation sites were further normalized for changes on the proteome level by dividing the site ratio by the corresponding protein group ratio. The normalization was done in R v. 4.1.2 (R Development Core Team (2012).

### Bioinformatics

Downstream statistical analysis of proteomics and phosphoproteomics was performed in R (version 3.6.0). The R package proteus (version 0.2.14) was used to analyze MaxQuant’s Proteomics output file “proteinGroups_SILAC.txt” and the phosphoproteomics output file “Phospho (STY)Sites.txt”. Differential expression (DE) analysis was performed with the R package Limma (version 3.42.2) outside of the package Proteus. As cut-off for statistical significance a multiple adjusted *p* value (p.adj value) < 0.05 was chosen, which is corrected for multiple testing to control the false discovery rate (FDR). In a first step the SILAC ratios were quantile normalized and log2 transformed. In order to identify differentially expressed proteins a linear model was then fitted to each protein/phosphosite as follows: exp =  ~ condition with “exp” representing expression of a protein and condition representing the ratios from fisetin treatment vs control. Using as null hypothesis log fisetin/control = 0 above formula was analyzed for the intercept term only, which is defined as the mean response value when all explanatory variables (here “condition”) were set to zero. For graphical visualization heatmaps and a volcano plot showing statistical significance -log10(*p*-value) versus log2 FC were produced. The same procedure was done for phosphosites and afterwards the phosphosite table was compared against the protein table to exclude phosphosites that were different due to differential expression at the protein level.

For a pathway and GO analysis in MDA-MB-468 cells, the g:Profiler tool (https://biit.cs.ut.ee/gprofiler/gost) (version e101_eg48_p14_baf17f0) was used. The protein IDs corresponding to the DE phosphosites and proteins with p.adj < 0.05 were copied into the g:Profiler tool. Homo sapiens was selected as the species and for the advanced options the following parameters were considered: only annotated genes, g:SCS threshold, 0.05 threshold and ENTREZGENE_ACC; before clicking on Run query. For GO analysis in MDA-MB-231 cells, DAVID 2021 (https://david.ncifcrf.gov/) was used. The protein IDs related to the DE phosphosites with significance B < 0.01 were copied into the DAVID tool. Homo sapiens was selected as the species and the most enriched pathways in DNA damage and repair pathways were selected and plotted.

### Statistics and densitometry

A densitometry analysis of the Western blots was performed by using LI-COR *Odyssey*® *Fc* with Image Studio Lite software version 5.2. (Bad Homburg v. d. Hoeh, Germany). Student’s *t*-test was applied to test a significant difference on the expected endpoint according to each experiment between two groups. The non-parametric Mann–Whitney U test was performed to analyze a significant difference on the number of chromosomal aberrations per metaphase induced by irradiation or fisetin treatment. *p* < 0.05 was considered statistically significant (* *p* < 0.05, ** *p* < 0.01, *** *p* < 0.001****, *p* < 0.0001).

## Results

### Inhibition of YB1 and AKT phosphorylation by fisetin is cell line dependent

Cell authentication confirmed the lack of ER and PR in all TNBC cell lines in comparison with the MCF-7 and T47D classified as ER+/PR+ (Fig. S1). In addition, it also suggested an association between the levels of phosphorylation of YB-1 (S102), RSK (T359/S363) and the expression of RSK2 (Fig. S1). Fisetin is a plant flavonoid with anticancer properties that inhibits RSK-mediated YB-1 S102 phosphorylation in the range of 20 to 80 µM by inhibiting the interaction of RSK1 and RSK2 to YB-1 in melanoma cells [16]. Here, we evaluated the effects of fisetin at the concentrations of 12.5, 25, 50 and 75 µM on phosphorylation of YB-1 (S102) and AKT (S473) in TNBC cells 24 h after treatment. We analyzed phosphorylation status of YB-1, AKT and RSK in TNBC cell lines; MDA-MB-231, MDA-MB-453, HS 578T and MDA-MB-468.

**Fig. 1:**
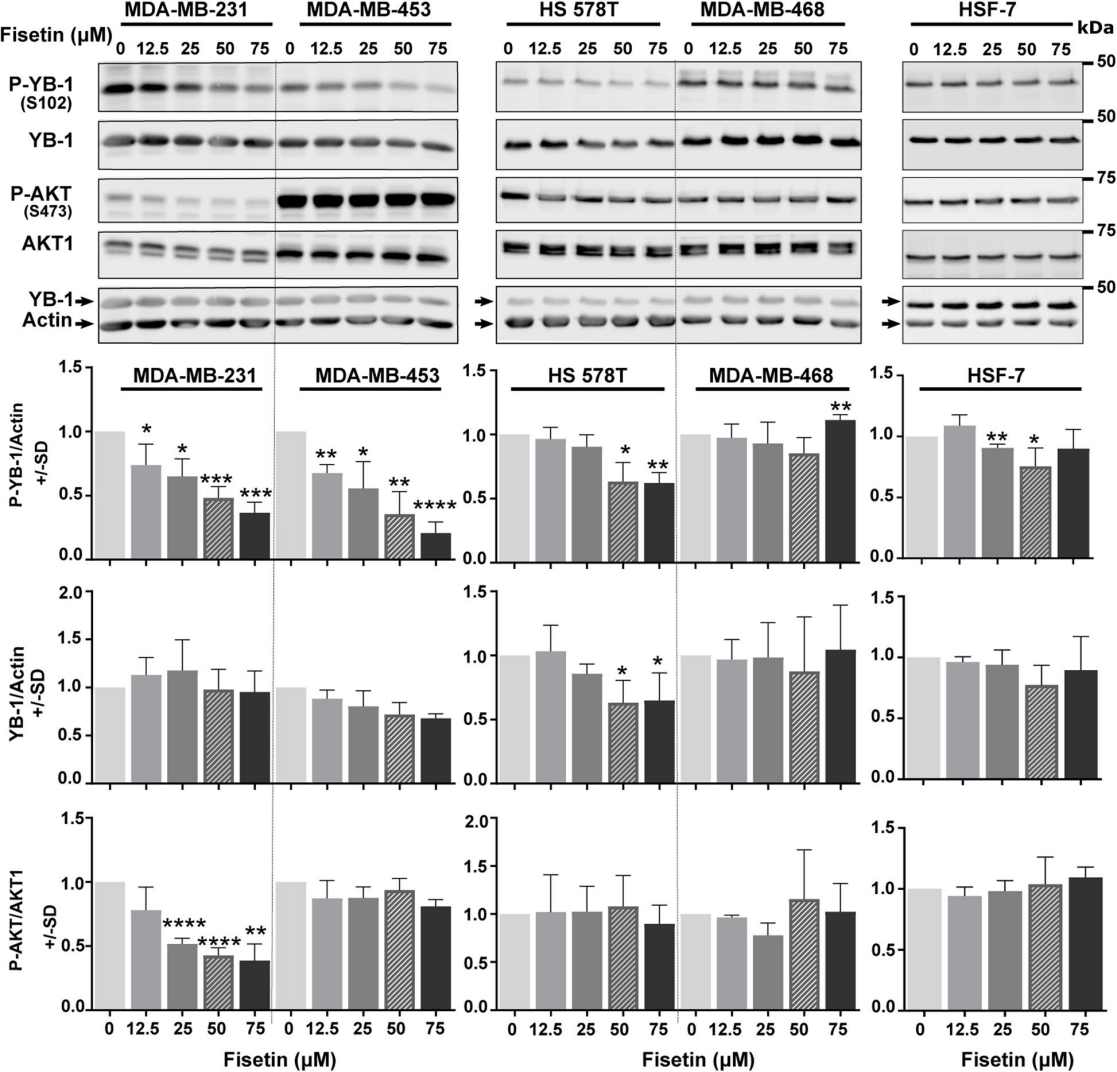
Effect of fisetin on phosphorylation of YB-1 and AKT is cell line dependent. The TNBC cells were treated with indicated concentrations of fisetin for 24 h. Thereafter, protein samples were extracted and loaded into a SDS-PAGE. The level of phosphorylation of YB-1 (S102) and AKT (S473) were detected by Western blotting. Blots were stripped and incubated with antibody against YB-1 and AKT1, respectively. Actin was detected from the YB-1 detected membrane without stripping as a loading control. The histograms represent the mean densitometry values ± SD of phospho-YB-1 to actin, YB-1 to actin and phospho-AKT to AKT1 normalized to 0 µM condition from 3 independent experiments. The asterisks indicate significant fisetin mediated changes on YB-1 and AKT phosphorylation (*p < 0.05, **p < 0.01, ***p < 0.001), ****p < 0.0001; students t-test). SD: standard deviation

Fisetin showed a cell line dependent inhibition effect (Fig. 1). It strongly reduced YB-1 (S102) phosphorylation in MDA-MB-231 and MDA-MB-453 cells in a dose- dependent manner without an effect on YB-1 protein expression. In HS 578T cells, fisetin reduced not only 50% YB-1 (S102) phosphorylation at the concentrations of 50 and 75 µM, but also YB-1 protein expression. In MDA-MB-468 cells, none of the fisetin concentrations inhibited YB-1 (S102) phosphorylation. Regarding AKT, fisetin failed to stimulate AKT (S473) phosphorylation in comparison to the RSK inhibitor LJI308 [6, 35] in all tested cell lines. However, it inhibited AKT (S473) phosphorylation in MDA-MB-231, with relatively lower levels of AKT phosphorylation shown in Fig. S1 and reported before [36]. In HSF-7 normal human fibroblast, fisetin slightly inhibited YB-1 (S102) phosphorylation only at concentrations of 25 and 50 µM (Fig. 1).

### Fisetin mimics RSK pharmacological inhibitors in terms of inhibiting YB-1 (S102) phosphorylation

**Fig. 2:**
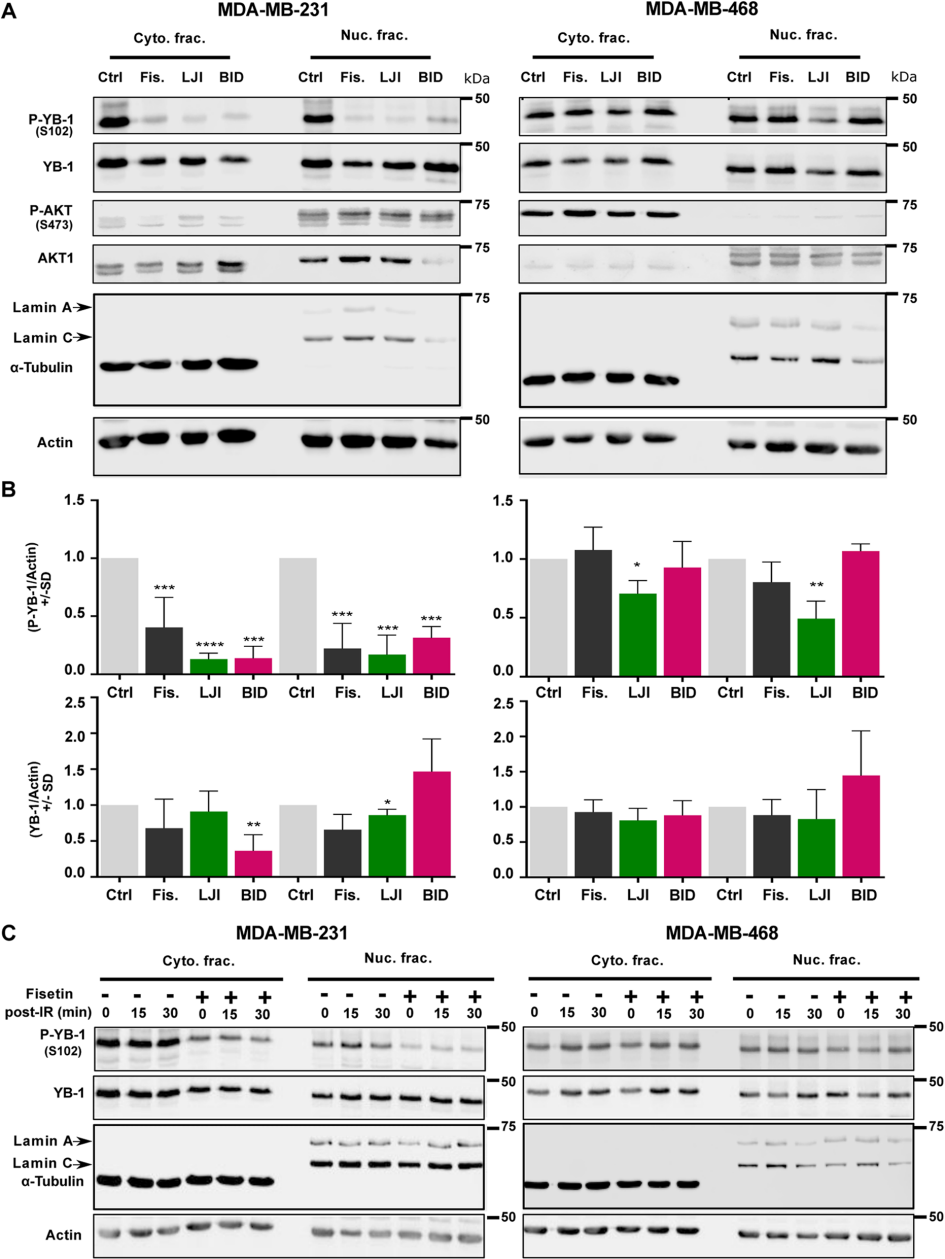
Fisetin and RSK inhibitors have similar effect on YB-1 phosphorylation. **A** The indicated cells were treated with fisetin (75 µM), LJI308 (LJI) (2.5 µM) or BI-D1870 (BID) (2.5 µM). Cytoplasmic and nuclear protein fractions were isolated after 24 h and subjected to SDS-PAGE. Phospho-YB-1, total YB-1, phospho-AKT and total AKT1 were detected by Western blotting. α-Tubulin and lamin A/C were used as cytoplasmic and nuclear markers, respectively. Actin was detected as loading control. **B** The histograms represent the mean densitometry values ± SD of P-YB-1 to actin and YB-1 to actin from 3 independent experiments normalized to DMSO treated control (Ctrl) condition. The asterisks indicate significant fisetin mediated changes on YB-1 phosphorylation (*p < 0.05, **p < 0.01, ***p < 0.001), ****p < 0.0001; students t-test). **C** Cells were treated with and without fisetin (75 µM, 24 h) and mock irradiated or irradiated 4 Gy. Cytoplasmic and nuclear protein fractions were isolated at the indicated times after IR and subjected to SDS-PAGE. Phospho-YB-1 and total YB-1 were detected by Western blotting. α-Tubulin and lamin A/C were used as cytoplasmic and nuclear markers, respectively. Actin was detected as loading control

Since fisetin inhibited YB-1 phosphorylation at S102 in MDA-MB-231 but not in MDA-MB-468 cells, we inquired whether this is due to the difference in inhibiting RSK activity or due to the divergent effects of fisetin on YB-1 (S102) phosphorylation. To this aim, we compared the effects of fisetin (75 µM, 24 h) with the those of two RSK pharmacological inhibitors LJI308 (LJI) and BI-D1870 (BID), both at concentrations of 2.5 µM administered for 24 h. The data shown in Fig. 2A and the related densitometry in Fig. 2B indicate that fisetin (75 µM) mimics two RSK inhibitors that could markedly inhibit YB-1 phosphorylation at S102 in both cytoplasmic and nuclear fractions of MDA-MB-231. Similar to the data shown in Fig. 1, fisetin did not inhibit YB-1 phosphorylation in cytoplasmic and nuclear fractions of MDA-MB-468. Interestingly and similar to the fisetin effect, both RSK pharmacological inhibitors inhibited YB-1 phosphorylation in MDA-MB-231 cells. In MDA-MB-468 cells, fisetin as well as the RSK inhibitor BID did not affect YB-1 phosphorylation. While LJI with a lower IC50 values [37] slightly reduced YB-1 phosphorylation in MDA-MB-468 cells in both cytoplasmic and nuclear fractions (Figs. 2A-B). This data indicates that RSK is one of the major targets of fisetin. It is known that IR, along with inducing DNA DSB, stimulates YB-1 phosphorylation in wild-type cells [5, 6]. In cells with mutations, e.g., gain of function mutation in *KRAS* or loss of function mutation in *PTEN*, YB-1 is highly phosphorylated and this is not further enhanced by IR [5, ]. Here, we inquired whether pattern of the effect of fisetin on YB-1 phosphorylation will be changed after irradiation in KRAS-mutated MDA-MB-231 and in PTEN-mutated MDA-MB-468 cells. As expected, both cell lines presented high level of YB-1 phosphorylation at S102, which was not further stimulated by IR (Fig. 2C). Intriguingly, the effect of fisetin on YB-1 phosphorylation at 15 min and 30 min after 4 Gy irradiation remained unchanged, *i.e.*, inhibited in MDA-MB-231 cells and not affected in MDA-MB-468 (Fig. 2C).

### Fisetin radiosensitizes TNBC cells

Inhibiting YB-1 phosphorylation on S102 by dual targeting of RSK and AKT was shown to be an efficient approach to block DSB repair and induce radiosensitization in breast cancer cells, independent of TNBC status [6]. We performed a clonogenic assay to investigate whether the inhibition of YB-1 phosphorylation by fisetin correlates with radiosensitization in the cell lines tested. To this aim, clonogenic assay was tested in 3 different combination settings, *i.e.*, one dose of IR (3 Gy) combined with multiple concentrations of fisetin (0 to 100 µM), multiple doses of IR (0 to 4 Gy) combined with one concentration of fisetin (75 µM) and fractionated irradiation (1 to 5 fractions of 1 Gy) combined with fractionated fisetin treatment (1 to 5 fractions of 75 µM). The data obtained from these experiments showed that fisetin induces radiosensitivity under all tested conditions. Fisetin at different concentrations induced radiosensitization in MDA-MB-231 cells irradiated with 3 Gy (Fig. S2A), in accordance with the inhibition of YB-1 phosphorylation shown in Fig. 1. Fisetin in non-irradiated cells (0 Gy) strongly inhibited clonogenic activity as well (Fig. S2A).

**Fig. 3:**
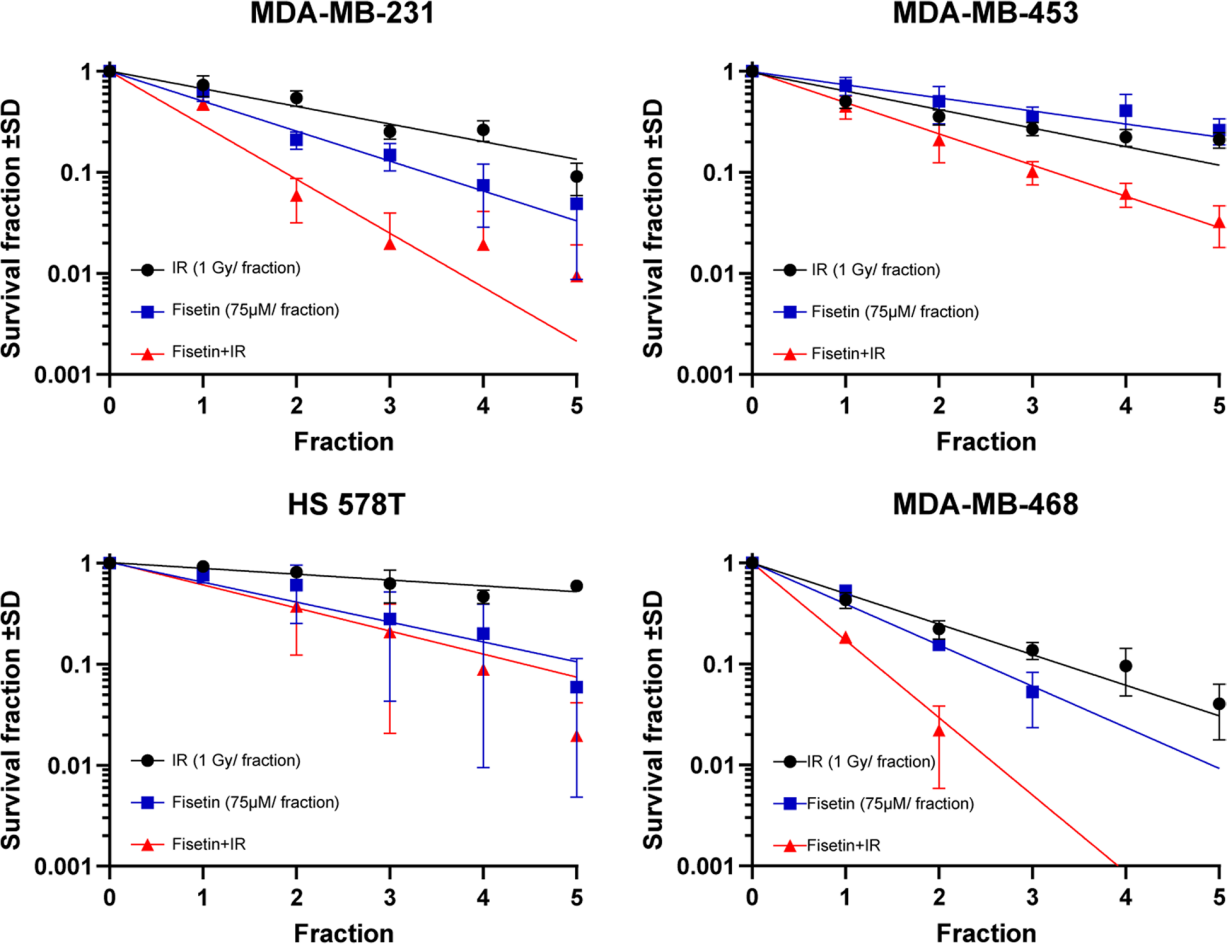
Fisetin radiosensitizes TNBC cells. The cells were treated with a vehicle (DMSO) or fisetin (75 µM) for 24 h, irradiated with a fractionated irradiation of 1 Gy and clonogenic assay was performed as described in the *Methods* section. The data points represent the mean surviving fraction ± SD of 12 data from 2 independent experiments (MDA-MB-231, MDA-MB-453, HS 578T) and 6 data from one experiment (MDA-MB-468)

In a separate experiment we compared the effect of fisetin (0 to 100 µM) with irradiation (0 to 4 Gy) in MDA-MB-231 and MDA-MB-468 on clonogenic activity and showed that, similar to IR, fisetin inhibits clonogenic activity in both of the cell lines tested (Fig. S2B). Based on this data in the further experiments we applied fisetin at 75 µM in combination with fractionated irradiation and investigated its radiosensitization in all 4 TNBC lines including MDA-MB-231 cells. Data shown in Fig. 3 indicates that fisetin induces radiosensitization in all TNBC lines, however the effect was cell line dependent.

Among the cell lines tested, HS 578T was the most radioresistant cell line and the radiosensitizing effect of fisetin started appearing in fraction 4. From this data we proposed that DSB repair machinery effectively repairs damages induced by 1 Gy fraction and that the potential radiosensitizing effect of fisetin might be observed when combined with a single dose of irradiation. We confirmed this hypothesis by performing a clonogenic assay in combination with fisetin (75 µM) and a single dose of irradiation of 0 to 4 Gy. Data obtained from this experiment confirmed the radiosensitizing effect of fisetin in HS 578T cells (Fig. S2C). Very interestingly, fisetin (75 µM) did not radiosensitize normal human fibroblast HSF-7 cells when combined with single dose irradiation 0 to 4 Gy (Fig. S2C).

### Fisetin blocks repair of IR-induced DSB

**Fig. 4:**
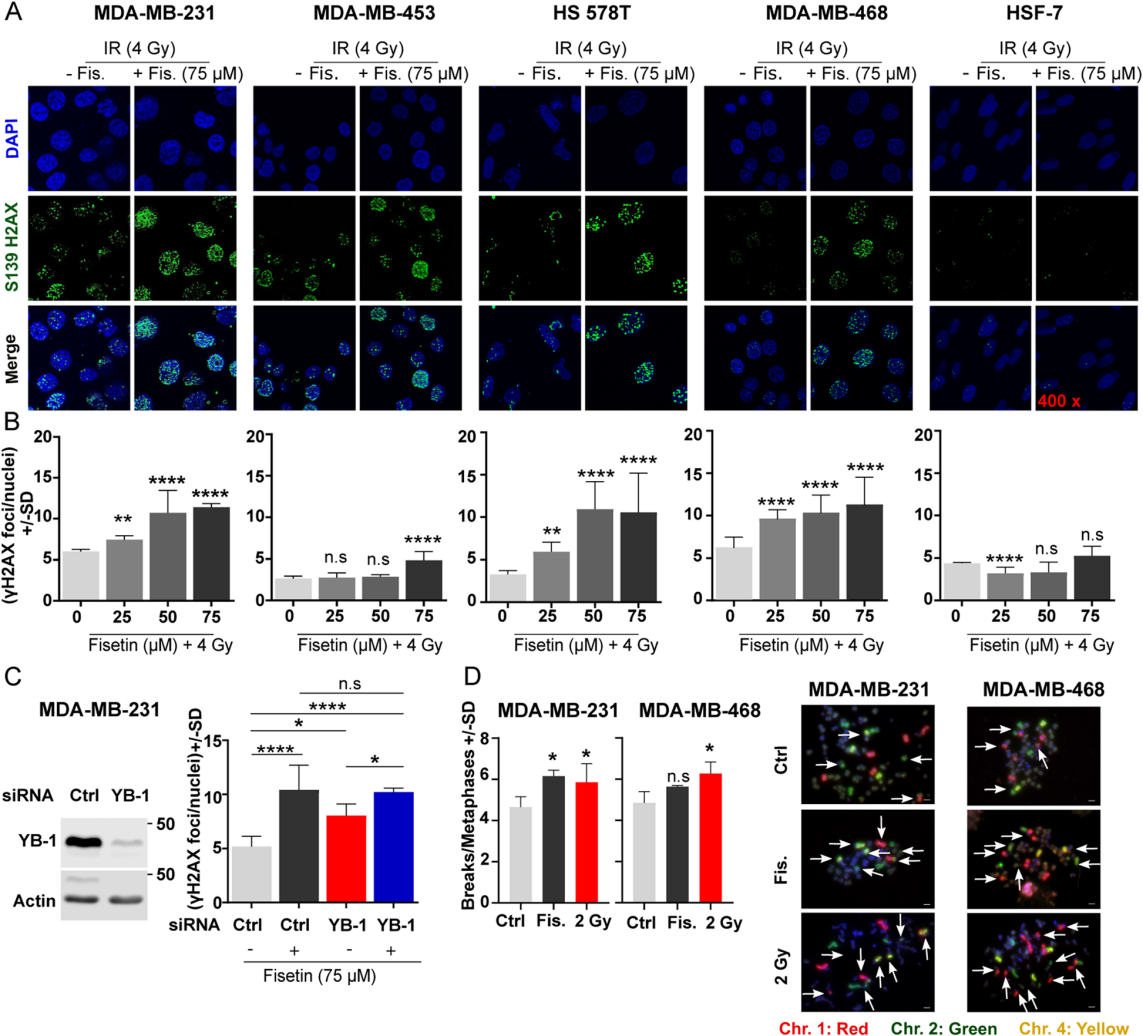
Fisetin inhibits the repair of IR-induced DSB, which leads to chromosomal aberration in TNBC cells. **A**, **B** The cells were treated with the indicated concentrations of fisetin for 24 h and irradiated with 4 Gy. Twenty-four hours after irradiation γH2AX foci assay was performed as described in the *Methods* section. **A** The representative immunofluorescent images of γH2AX foci used for analyses. **B** Asterisks indicate significant inhibition of DSB repair shown by increased mean residual γH2AX ± SD after fisetin treatment compared to DMSO treated/ 4 Gy irradiated control (Ctrl) condition in 500 nuclei in MDA-MB-231 cells, 700 nuclei in MDA-MB-453 cells, 600 nuclei in MDA-MB-468 cells, 500 nuclei in HS 578T cells and 440 nuclei in HSF-7 cells, from 3 independent experiments. **C** MDA-MB-231 were transfected with 50 nM indicated siRNA and protein samples were isolated 72 h after transfection to analyze knockdown efficiency by Western blotting. In the parallel cultures, 24 h after transfection cells were treated with DMSO or fisetin (75 µM) for additional 24 h and irradiated with 4 Gy. γH2AX was performed 24 h after irradiation (72 h after transfection) and counted using FoCo software. The asterisks indicate significant difference in mean γH2AX ± SD between the indicated conditions (*p < 0.05, **p < 0.01, ***p < 0.001), ****p < 0.0001; students t-test) analyzed in 444 cells, from 3 independent experiments. The DMSO concentration in the cells treated with different concentrations of fisetin was kept similar. **D** The mean number of chromosomal aberrations analyzed in at least 50 metaphases 48 h after irradiation (2 Gy) or 72 h after treatment with fisetin (75 µM). (*p < 0.05, Mann–Whitney U test): n.s. = non-significant. Chr.: Chromosome

It is known that YB-1 stimulates repair of IR-induced DSB [5, 6]. Thus, we investigated whether fisetin affects the repair of DSB in association with the inhibition of YB-1 phosphorylation at S102. Analyzing residual DSB 24 h after irradiation revealed that the fisetin pretreatment at concentrations of 25, 50 and 75 µM for 24 h inhibits repair of DSB in all 4 TNBC cell lines tested, independent of its effect on YB-1 phosphorylation at S102 (Fig. 4A-B). Fisetin (75 µM) did not affect IR-induced DSB repair in HSF-7 cells, while at the concentration of 25 µM it stimulated DSB repair (Fig. 4A-B), which is in favor of future clinical applications of the drug. To analyze whether induction of damage is different in the presence and absence of fisetin, the number of γH2AX foci was analyzed 30 min after irradiation with 1 Gy in cells pretreated with and without fisetin (75 µM). The data presented in Fig. S3A indicates that the frequency of γH2AX foci in fisetin treated cells is higher than in control cells at either the 0 Gy or 1 Gy irradiation condition, which indicates that fisetin can induce DSB. Functions of fisetin as an inhibitor of the repair of IR-induced DSB and as an inducer of DSB when applied as a single treatment was tested in MDA-MB-231 and MDA-MB-468 cells after treatment with 0, 25, 50 and 75 µM for 48 h. Data shown in Fig. S3B indicates that fisetin at a concentration of 75 µM induced DSB in both cell lines. However, fisetin at lower concentrations, *i.e.,* 25 and 50 µM did not induce DSB in MDA-MB-231 cells. The frequency of residual DSB was higher when these concentrations of fisetin were combined with IR, indicating inhibition of repair of IR-induced DSB (Fig. 4A-B). By applying YB-1-siRNA in MDA-MB-231 cells, we were able to show that the inhibitory effect of fisetin on DSB repair is much stronger than the effect of YB-1 knockdown (Fig. 4C). These data indicate that fisetin blocks the repair of IR-induced DSB and that this effect is only partially dependent on YB-1.

Residual DSB in proliferating cells result in a variety of chromosomal aberrations that lead to cell death. We investigated whether fisetin induces chromosomal aberration in MDA-MB-231 and MDA-MB-468 cells, in which YB-1 phosphorylation was differentially affected by fisetin, *i.e.*, inhibited in MDA-MB-231 cells and not affected in MDA-MB-468 cells. Fisetin (75 µM, 72 h) induced chromosomal aberration in MDA-MB-231. In MDA-MB-468 cells, the frequency of aberration was also slightly but not significantly enhanced (Fig. 4D). In both cell lines, IR (2 Gy) induced chromosomal aberration (Fig. 4D).

### Fisetin inhibits DSB repair through interference with C-NHEJ and HR repair pathways

**Fig. 5:**
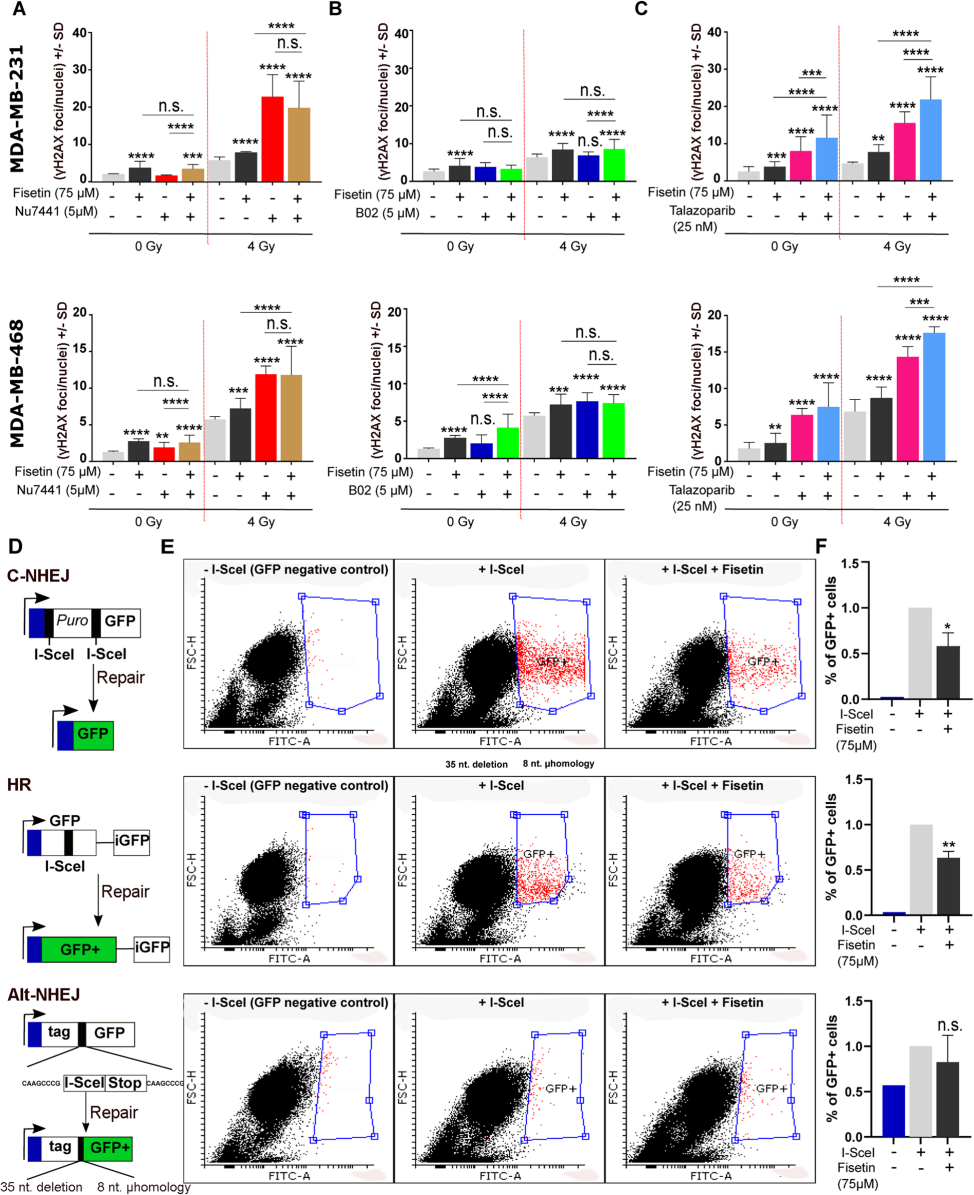
Fisetin inhibits DSB through the HR and C-NHEJ repair pathways. **A**-**C** MDA-MB-231 and MDA-MB-468 cells were treated with or without fisetin (75 µM) for 22 h and followed by treatment with or without DNA-PKcs inhibitor (NU7441, 5 µM), Rad51 inhibitor (B02, 5 µM) or PARP inhibitor (Talazoparib, 25 nM) for 2 h. The DMSO concentration in cells treated with different inhibitors was kept similar. Thereafter, cells were mock irradiated or irradiated 4 Gy and γH2AX was performed 24 h after IR. γH2AX foci per nuclei were counted using FoCo software. The data are presented as the mean number of foci per nuclei ± S.D. The asterisks indicate significant inhibition of DSB repair shown by increased mean residual γH2AX ± SD after treatment with indicated inhibitors compared to DMSO treated/ 4 Gy irradiated control condition or compared between indicated groups from at least 300 nuclei in MDA-MB-231 cells and MDA-MB-468 cells from at least 3 independent experiments (*p < 0.05, **p < 0.01, ***p < 0.001), ****p < 0.0001; students t-test). **D** U2OS cells harboring different DNA repair constructs including HR, C-NHEJ and Alt-NHEJ were used. **E** The cells were either transiently transfected with an inducible endonuclease I-SceI plasmid (800 ng/ml) or not transfected as a negative control. Twenty-four hours after transfection, cells were treated with or without fisetin (75 µM, 24 h). Nuclear translocation of I-SceI was induced by 100 ng/ml triamzinolonacetonid and twenty-four hours later the percentage of GFP positive cells were determined using FACS. **F** The bar graphs show the mean percentage of GFP positive cells ± SD from 4 independent experiments normalized to DMSO treated control condition. Asterisks indicate inhibition of the indicated repair pathway by fisetin treatment (*p < 0.05, **p < 0.01; students t-test). The data shown for GFP-negative control cells is the mean from 2 independent experiments

IR-induced DSB are repaired either by C-NHEJ or Alt-NHEJ throughout the cell cycle and by HR during the S and G2 phases. We investigated which DSB repair pathway was inhibited by fisetin by combining fisetin with a specific inhibitor of each repair pathway. To this end, DNA-PKcs inhibitor NU7441 (5 µM), Rad51 inhibitor B02 (5 µM) and PARP inhibitor Talazoparib (25 nM) were used as the inhibitors of C-NHEJ, HR and Alt-NHEJ repair pathways, respectively. Data shown in Fig. 5A revealed that treatment with fisetin and NU7441 significantly inhibited DSB repair in MDA-MB-231 and MDA-MB-468 cells after 4 Gy irradiation. B02 inhibited DSB repair in MDA-MB-468 but not in MDA-MB-231 cells (Fig. 5B). A combination of fisetin neither with Nu7441 (Fig. 5A) nor B02 (Fig. 5B) enhanced residual DSB compared to single treatments. Similar to fisetin, talazoparib as a PARP inhibitor, induced DSB as monotherapy and inhibited repair of IR-induced DSB in both cell lines (Fig. 5C). A combination of fisetin with talazoparib resulted in an additive effect after irradiation in both cell lines (Fig. 5C).

To support the data by pharmacological inhibitors of the specific DSB repair pathways, the effect of fisetin on I-SceI-induced DSB was tested in osteosarcoma cell lines U2OS cells harboring reporter constructs specific for the individual repair pathways. Schematic figures demonstrating the constructs for the repair pathways they report have been outlined in Fig. 5D-F. The data obtained using these cells indicate that pretreatment with fisetin (75 µM) inhibits I-SceI-induced DSB repair in cells reporting HR and C-NHEJ but not Alt-NHEJ as shown by the FACS plots (Fig. 5E) as well as the mean percentage of GFP positive cells (Figs. 5F).

### Effect of fisetin in combination with IR on apoptosis and autophagy

**Fig. 6:**
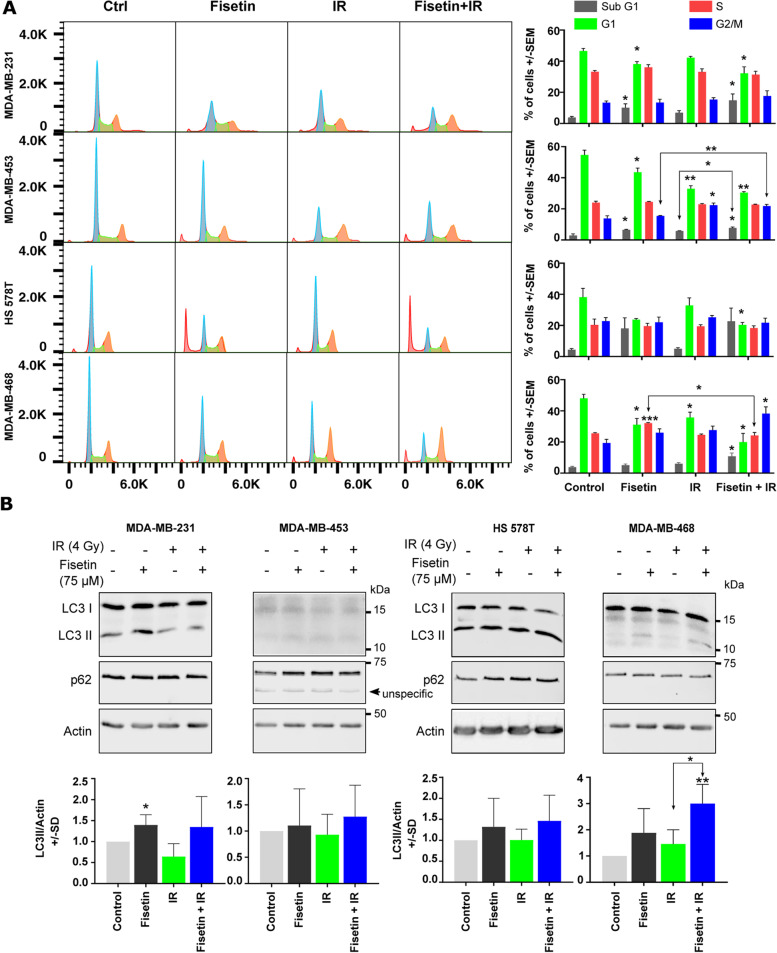
Fisetin in combination with irradiation does not affect cell cycle progression and autophagy. **A** log-phase cells were treated with fisetin (75 µM) for 24 h and irradiated with 4 Gy. Forty-eight hours after irradiation, cells were collected and fluorescence-activated cell sorting analysis was performed as described before [27]. The percentage of cells in different cell cycles as mean ± SD was calculated from at least 3 independent experiments and graphed. The asterisks indicate significant differences in individual treatment groups compared to the DMSO treated control (Ctrl) condition or between the arrows indicated conditions (*p < 0.05, **p < 0.01, ***p < 0.001; students t-test). **B** The cells were treated with a vehicle or fisetin (75 µM) for 65 h and mock irradiated or irradiated with 4 Gy. Protein samples were isolated 7 h after irradiation and subjected to SDS-PAGE. The level of LC3I/II and p62 were detected using Western blotting. The experiments were repeated at least 3 times and similar results were obtained. Actin was detected as the loading control. Bar graphs represent the mean densitometry of LC3-II to actin from at least 3 independent experiments normalized to 1 in control condition. The asterisks indicate significant differences in mean LC3-II/actin between the indicated conditions or compared to untreated control (*p < 0.05, **p < 0.01; students t-test)

As an alternative to residual DSB mediated cell death by mitotic catastrophe, enhanced apoptosis by the combination of fisetin and radiation might be a potential mechanism of radiosensitization by fisetin. Here, using flowcytometry analysis, it was shown that fisetin (75 µM) treatment for 72 h (24 h before and 48 h after IR) generally reduces the percentage of cells in G1 phase (Fig. 6A). Fisetin significantly enhanced sub-G1 population as an indication of apoptosis only in MDA-MB-231 and MDA-MB-453 cells. Radiation (4 Gy) did significantly reduce the G1 population only in MDA-MB-453 and MDA-MB-468 cells. Irradiation did not induce apoptosis in either of 4 TNBC cell lines. Interestingly, a combination of fisetin with IR enhanced apoptosis compared to fisetin or radiation alone only in MDA-MB-468 cells (Figs. 6A).

Controversial data has been reported regarding the effect of fisetin on autophagy. It is not known how the level of autophagy is changed in TNBC cells after a fisetin treatment in combination with irradiation. In this study, we investigated if there was a correlation between the expression pattern of LC3-II and p62 as autophagy markers and radiosensitizing effect of fisetin. Our data demonstrated that fisetin (75 µM, 72 h) markedly induced the expression of LC3-II in MDA-MB-231 and MDA-MB-468 cells without changes on the expression level of p62. Neither IR nor the combination of IR with fisetin induced the expression of autophagy markers, as shown by Western blotting (Fig. 6B). However, similar to a 24 h treatment, treatment with fisetin for 72 h induced radiosensitization in MDA-MB-231 cells when combined with single dose irradiation of 3 Gy (Fig. S2A) or IR doses of 1 to 4 Gy (data not shown). Together, the data presented for DSB repair, autophagy and apoptosis indicates that the combination of fisetin with radiation leads to radiosensitization due to enhanced residual DSB that results in mitotic catastrophe but not stimulating apoptosis or regulating autophagy in TNBC cells.

### Fisetin modulates activation of DDR signaling cascades

**Fig. 7:**
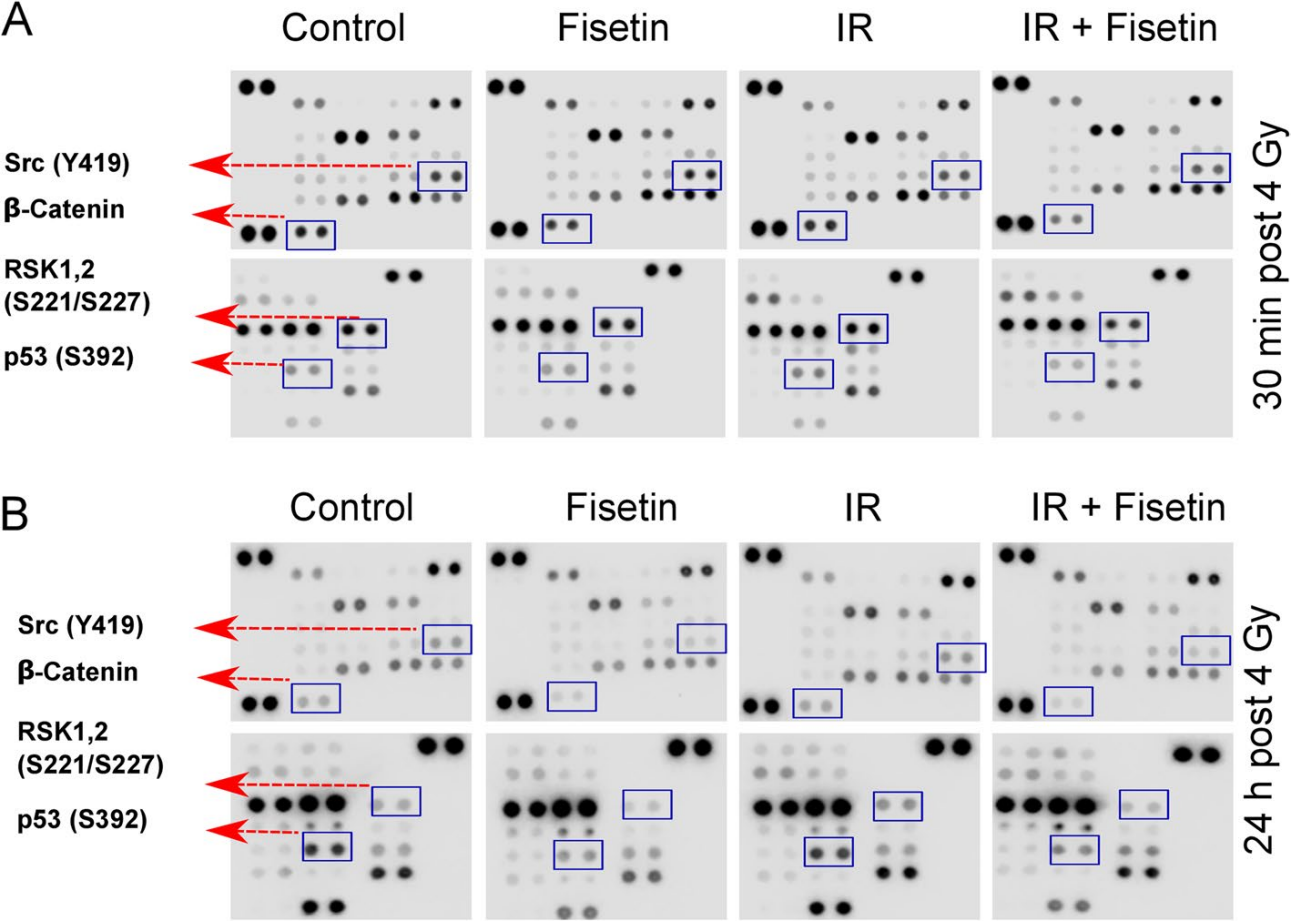
Effect of fisetin om multiple Kinases in MDA-MB-231 cells after irradiation. MDA-MB-231 cells were treated with fisetin (75 µM) for 72 h and irradiated with 4 Gy. Protein samples were isolated either 30 min (**A**) or 24 h after irradiation (**B**). A phospho-kinase proteome analysis was performed according to the manufacturer’s protocol after the described treatments

Fisetin in the range of 20 to 80 µM interacts with RSK1 and RSK2 to inhibits YB-1 phosphorylation at S102 in melanoma cells [16]. By applying a short scale phospho-kinase array in irradiated MDA-MB-231 cells we could show that fisetin (75 µM) inhibits phosphorylation of RSK1/2 (S221/S227) as the major kinases involved in YB-1 phosphorylation. However, fisetin in combination with IR markedly inhibited p53 phosphorylation (S392), phosphorylation of Src kinase (Y419) and suppressed the expression of ß-catenin, analyzed at 30 min as well as 24 h post-IR (Fig. 7). The inhibitory effect of fisetin alone in non-irradiated condition in the 30-min post-IR experiment was mild. This data indicated that fisetin may affect multiple pathways involved in cell survival.

**Fig. 8:**
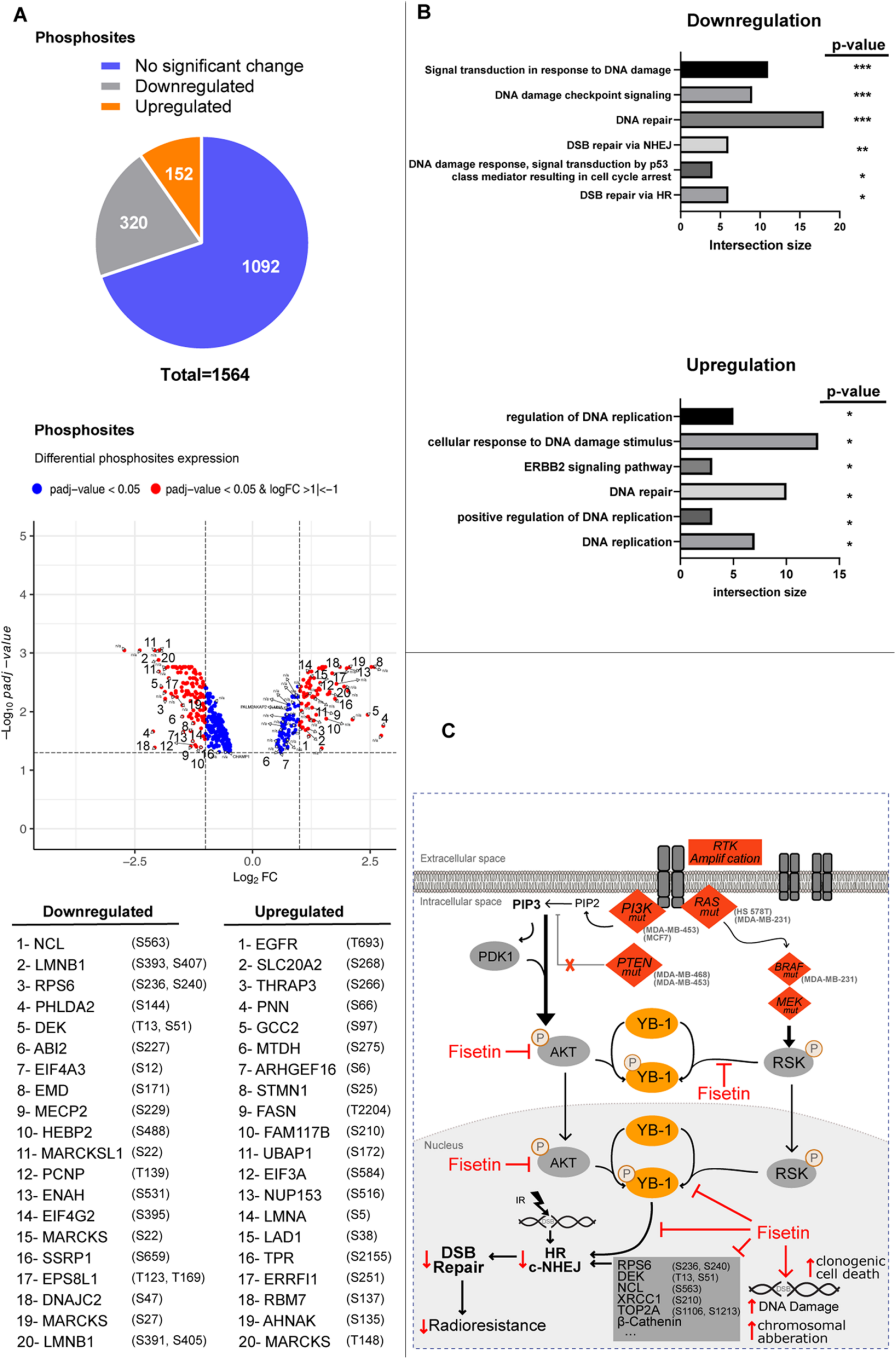
DDR are the major target of fisetin in irradiated cells. A phosphoproteomic study was performed in MDA-MB-468 cells, treated with or without fisetin (75 µM) for 24 h and irradiated with 4 Gy. Thirty minutes after irradiation cells were isolated and phosphoproteomics was performed as described in the *Material & Methods* section. **A** A total of 472 deregulated phosphosites (320 downregulated and 152 upregulated) and 47 deregulated total proteins were identified. **B** The gene ontology analysis indicates that phospho-proteins involved in DDR are the most frequently downregulated ones by fisetin in irradiated cells. The asterisks indicate significant deregulation (*N* = 3, *p < 0.05, **p < 0.01, ***p < 0.001; values extracted from g:profiler analysis). **C** The proposed signaling pathway targeted by fisetin to interfere with repair of IR-induced DS

To analyze a possible effect of fisetin on DDR signaling, a large scale phosphoproteomic study was performed in fisetin pre-treated and irradiated cells compared to irradiation alone. In MDA-MB-468 cells, in which fisetin does not inhibit YB-1 phosphorylation, a total of 472 phosphosites from 1564 analyzed phosphosites were found to be up- or down regulated (Fig. 8A, Fig. S4). DEK (T13, S51), nucleolin (NCL) (S563), XRCC1 (S210) and TOP2A (S1106) were among the top 10 inhibited phosphorylation sites involved in DNA repair. Next, we performed a gene ontology (GO) enrichment analysis to verify if deregulated phosphosites are involved in DDR signaling, *i.e.*, DSB repair. The GO data presented in Fig. 8B and Table S1 indicates that gene products involved in DSB repair are among the most frequently inhibited targets participating in DDR signaling. Interestingly, the GO biological process analysis in irradiated MDA-MB-231 cells presented in Fig. S5 was similar to the data obtained in MDA-MB-468 cells indicating the importance of DNA repair gene products as the most important targets of fisetin in irradiated cells (Fig. S5).

## Discussion

TNBC is an aggressive type of breast cancer with poor outcomes. Beside surgery, radiotherapy is the main treatment option for TNBCs; unfortunately, radioresistance frequently occurs and diminishes the results of the therapy outcome. YB-1 as a multi-functional oncoprotein is overexpressed in different tumor types and plays a pivotal role in cell death resistance mechanisms. Fisetin is a plant flavonoid compound that interferes with RSK mediated YB-1 activity. In this study, we uncovered potential targets of fisetin in TNBC cells and investigated effect of fisetin on DSB repair and radiation response. The data obtained demonstrates that fisetin induces radiosensitization in association with inhibiting DSB repair in TNBC cells but not in normal human skin fibroblast. Fisetin inhibited DSB repair by inhibiting the HR and C-NHEJ repair pathways.

### Fisetin interferes with the DDR signaling RSK and AKT pathways

RSK and AKT are the main kinases phosphorylating YB-1 at S102 [38], prerequisite for the effect of YB-1 in stimulating repair of IR induced DSB [6]. AKT, besides its role in YB-1 phosphorylation, stimulates repair of IR-induced DSB by directly binding to DNA-PKcs [27, 39, 40]. Thus, due to the compensatory role of AKT, inhibiting YB-1 activity by solely targeting RSK is not an effective approach to inhibit DSB repair and induce radiosensitization [6]. To this aim, it was previously shown that co-targeting of RSK and AKT was more effective than single targeting of each kinase in terms of inhibiting cell proliferation [35], blocking DSB repair and inducing chemosensitivity [35] as well as radiosensitivity [6]. TNBC cell lines in this study harbor the key mutations (Table S2) that stimulate the PI3K/AKT and MAPK/RSK pathways, as underlying pathways involved in phosphorylation of YB-1 at S102. Analyzing basal phosphorylation of YB-1, AKT and RSK and the expression of RSK1 and RSK2 revealed that phosphorylation of YB-1 at S102 is mainly associated with the expression of RSK1 and RSK2 as well as the phosphorylation of RSK (T359/S363) (see Fig. S1). RSK and YB-1 are highly phosphorylated in MDA-MB-231 and MDA-MB-468 cells while MDA-MB-453 and HS 578T cells present high phosphorylation of AKT (S473) and low phosphorylation of YB-1. According to this data, phosphorylation of YB-1 is mainly stimulated by RSK, suggesting that RSK targeting is necessary to efficiently inhibit YB-1 phosphorylation and YB-1-dependent cellular functions. Fisetin is known to block YB-1 phosphorylation in melanoma cells by binding mainly to RSK2 and to a lesser degree to RSK1 [16]. Likewise, fisetin inhibits the PI3K/AKT pathway in breast cancer cells [18]. In the present study, we applied fisetin as an alternative approach to AKT/RSK dual targeting strategy to block YB-1 phosphorylation. Our data in TNBC cells supports the reported effect of fisetin on YB-1 phosphorylation in melanoma cells [16]. Fisetin mimicked the effect of RSK inhibitors on YB-1 phosphorylation, indicating that fisetin interfered with RSK in TNBC cells. It inhibited YB-1 phosphorylation in 3 out of the 4 TNBC cell lines tested. In MDA-MB-468 cells, in which fisetin did not affect YB-1 phosphorylation at S102, the lack of an effect was also observed by two well-described RSK inhibitors, in the presence or absence or irradiation. This set of data indicates that S102 phosphorylation of YB-1 in MDA-MB-468 cells is mainly RSK independent. In line with this conclusion, MDA-MB-468 cells lack the expression of PTEN [41], which results in hyperactivation of AKT and, consequently, AKT-dependent YB-1 phosphorylation [6].

### Fisetin induces DSB and interferes with DSB repair after IR

Nuclear localization of YB-1 is linked to a poor prognosis in different tumors, as reviewed elsewhere [38]. Binding of YB-1 to DNA repair proteins MSH2, DNA polymerase delta, Ku80 and WRN proteins has been described before [13]. In this context, our previous studies demonstrated that a genetic knockdown of YB-1 or blocking S102 phosphorylation of YB-1 using a specific peptide impairs the repair of IR-induced DSB [5, 6]. Here, we could show that fisetin inhibits YB-1 phosphorylation at S102 in 3 out of 4 TNBC cell lines tested, indicating the potential of fisetin to block the repair of DSB after irradiation. Interestingly, in all cell lines tested, including MDA-MB-468 cells, which lack a response to the effect of fisetin on YB-1 S102 phosphorylation, the frequency of residual DSB shown by γH2AX was enhanced in the combination of fisetin with IR compared to IR alone. This effect was associated with radiosensitization in all tumor cells tested but not in normal fibroblasts. As expected, fisetin did not affect DSB repair in normal fibroblasts. In line with this observation, Piao et al. reported that fisetin has a protective effect against γ-irradiation-induced oxidative stress and cell damage in Chinese hamster lung fibroblasts [42]. Interestingly, fisetin alone was shown to function as a DNA damage inducing agent when applied at 75 µM in all TNBC cells tested. This effect of fisetin in TNBC cells correlates with the previous reports from other laboratories indicating DNA damage induced in three tumor entities by fisetin at different concentrations, *i.e.*, in hepatic cancer (60 µM) [43], gastric cancer (50 µM) [44] and pancreatic cancers (50 and 100 µM) [17]. In the current study, applying fisetin at the concentrations of 25 and 50 µM did not induce DNA damage as shown in MDA-MB-231 (Fig. S3) but inhibited repair of IR-induced DSB at these as well as a concentration of 75 µM (Fig. 4). This data supports the effect of fisetin on DDR signaling. Thus, fisetin can potentially function as a double-edged sword in irradiated TNBC cells. Similar to IR, it functions as a DSB inducing agent, but simultaneously inhibits repair of DSB that are induced by IR. At present, it is not known whether the quality and complexity of DSB induced by fisetin and IR are similar. This is a topic that remains to be investigated.

DSB is the major type of DNA damage involved in radiotherapy-induced cell death. Activation of signaling pathways such as RSK/YB-1 and PI3K/AKT by irradiation stimulates DSB repair and diminishes the effect of radiotherapy. [6, 39]. So far existing data indicates that fisetin inhibits survival signaling pathways in different tumors, e.g., RSK/YB-1 in melanoma [16], PI3K/AKT in pancreatic cancer [45] and YB-1 in TNBC cells as shown in the present study. The inhibition of prosurvival pathways by fisetin contrasts with the effect of IR, which is known to stimulate these pathways [5, 6, 46]. Thus, fisetin treatment is expected to block clonogenic activity. Comparing the effect of single doses of fisetin with the effect of single doses of IR on clonogenic activity of MDA-MB-231 and MDA-MB-468 cells supports this conclusion (Fig. S2B). The anti-clonogenic activity of fisetin in MDA-MB-468 cells was stronger compared to that in MDA-MB-231 cells. This might be due to the stronger effect of fisetin in DSB induction in MDA-MB-468 compared to that in MDA-MB-231 cells (Fig. S3B). Our results from the present study indicate that fisetin in combination with IR inhibits the major DSB repair pathway, *i.e.*, C-NHEJ and HR (see Figs. 5). The data on the effect of fisetin on HR is also supported by a recent report from Huang et al., who showed that HR repair efficiency in pancreatic cancer cells is diminished by fisetin treatment [47]. In this study, the authors demonstrate that fisetin inhibiting HR is due to a modification of N6-methyladenosine [47]. However, gene ontology from our phosphoproteomic study revealed that a variety of phosphosites involved in DDR are inhibited by fisetin treatment in irradiated cells. Among them, gene products involved in DNA repair, chromatin binding and DNA replication are the most affected. DEK (T13, S51) [48], nucleolin (S563) [49], XRCC1 (S210) [50] and TOP2A (S1106) [51] were found to be among the top 20 fisetin inhibited phospho-proteins that have been reported to be involved in DDR signaling*, i.e.,* DSB repair. Thus, we suggest that fisetin affects DDR at multiple levels rather than blocking it at a specific stage by affecting/inhibiting a single target.

### Radiosensitization of TNBC by fisetin results from boosting DSB but not modulating autophagy and apoptosis

Non-repaired DSB leads to cell death by different mechanisms, *i.e.,* mitotic catastrophe, apoptosis and autophagy. So far, published data indicates that fisetin induces cell death by stimulating apoptosis. Here, we were able to show a significant enhancement of apoptosis by fisetin treatment in two out of the 4 TNBC cell lines. IR did not induce apoptosis in any of the cell lines tested. Importantly, a combination of fisetin with radiation slightly stimulated apoptosis in only one cell line (MDA-MB-468). However, the radiosensitizing effect of fisetin was observed in all TNBC cells but not in normal human fibroblast. The lack of enhanced apoptosis in TNBC cells after combination of fisetin with irradiation is in conflict with the report by Chen et al*.,* who described that fisetin radiosensitizes *TP53* mutated HT-29 colorectal cancer cells through stimulating apoptosis [52]. The initiation of apoptosis or alternative types of cell death depends on a variety of parameters. Complexity of DSB, expression of the components of the underlying signaling pathways involved in a certain type of cell death and the mutation status of these components are the most crucial parameters, which vary in different cell lines. Thus, the conflicting results might be due to the different in cell lines investigated. Although, all the TNBC cell lines tested so far in our study are mutated in *TP53* and radiosensitized by fisetin, *TP53* mutation is not a prerequisite for fisetin-mediated radiosensitization since fisetin did not radiosensitize the *TP53* mutated non-TNBC cell line T74D (data not shown). Additionally, fisetin has been reported to improve radiotherapy outcome of *TP53* wild-type CT‑26 xenograft tumors [53]. Thus, this data may indicate that the radiosensitizing effect of fisetin in breast cancer cells is dependent on the TNBC status, *i.e.*, the expression of HER2, estrogen and progesterone receptors. Radiation induces autophagy in breast cancer cells [54] however contradictory results exist in terms of the effect of autophagy on post-irradiation cell survival. It has been shown that the inhibition of autophagy by pharmacological inhibitors radiosensitizes breast cancer cells, liver cancer cells and esophageal squamous cell carcinomas [54–56]. In contrast, a genetic knockdown of ATG5 and Beclin 1 was shown to mediate radioresistance in prostate cancer cells [57]. The differential effect of fisetin on autophagy has been reported as well. The induction of autophagy by fisetin was described in pancreatic cancer cells [58], whereas fisetin inhibited autophagy in hepatocellular carcinoma HepG2 cells [19]. Independent of the function of autophagy in post-irradiation cell survival, as well as the effect of fisetin on autophagy induction, in the present study we could show that fisetin in combination with radiation does not change autophagy levels when compared to radiation alone.

## Conclusion

The application of fisetin may improve the radiotherapy outcome of TNBC patients through interference with signaling pathways involved in DSB repair (Fig. 8C) and consequently mitotic catastrophe.

## Supplementary Information

**Additional file 1: Table S1. **The GO indicating modified gene products involved in DSB repair.

**Additional file 2: Table S2. **Mutation status on KRAS , HRAS, PTEN and TP53 in the TNBC cell lines under study.

**Additional file 3: Fig. S1** The pattern of expression of the estrogen-, progesterone- and HER2 receptors, as well as the activation status of YB-1, AKT and RSK in the indicated cell lines under study. **Fig. S2** The radiosensitizing effect of fisetin in combination with single dose irradiation. **Fig. S3** Frequency of DSB induction after fisetin, IR and the combination of fisetin and ITR. **Fig. S4** Heat map, gene ontology and pathway analysis of phosphosites in MDA-MB-468 cells. **Fig. S5** Gene ontology analysis of MDA-MB-231 after pretreatment with fisetin.

## Data Availability

All data in our study are available upon request.

## Abbreviations

A-NHEJ: Alternative non-homologous end joining
BID: BI-D1870
C-NHEJ: Classical non-homologous end joining
CSD: Cold-shock domain
DDR: DNA damage response
DE: Differential expression
DSB: Double-strand breaks
ER: Estrogen receptor
GFP: Green fluorescence protein
GO: Gene ontology
Gy: Grey
HR: Homologous recombination
IR: Ionizing radiation
LJI: LJI308
PE: Plating efficiency
PR: Progesterone receptor
SF: Survival fraction
SILAC: Stable Isotope Labeling by Amino Acids in Cell Culture
TA: Triamcinolonacetonid
TNBC: Triple-negative breast cancer
YB-1: Y-box binding protein-1
