# Supplementary material for: Fisetin induces DNA double-strand break and interferes with the repair of radiation-induced damage to radiosensitize triple negative breast cancer cells
Source: J Exp Clin Cancer Res. 2022 Aug 22;41:256. doi: 10.1186/s13046-022-02442-x (PMC9394010; doi:10.1186/s13046-022-02442-x)
Supplement: Supplementary file 3 — Additional file 3: Fig. S1 The pattern of expression of the estrogen-, progesterone- and HER2 receptors, as well as the activation status of YB-1, AKT and RSK in the indicated cell lines under study. Fig. S2 The radiosensitizing effect of fisetin in combination with single dose irradiation. Fig. S3 Frequency of DSB induction after fisetin, IR and the combination of fisetin and ITR. Fig. S4 Heat map, gene ontology and pathway analysis of phosphosites in MDA-MB-468 cells. Fig. S5 Gene ontology analysis of MDA-MB-231 after pretreatment with fisetin. [file 13046_2022_2442_MOESM3_ESM.pptx]

## Slide 1
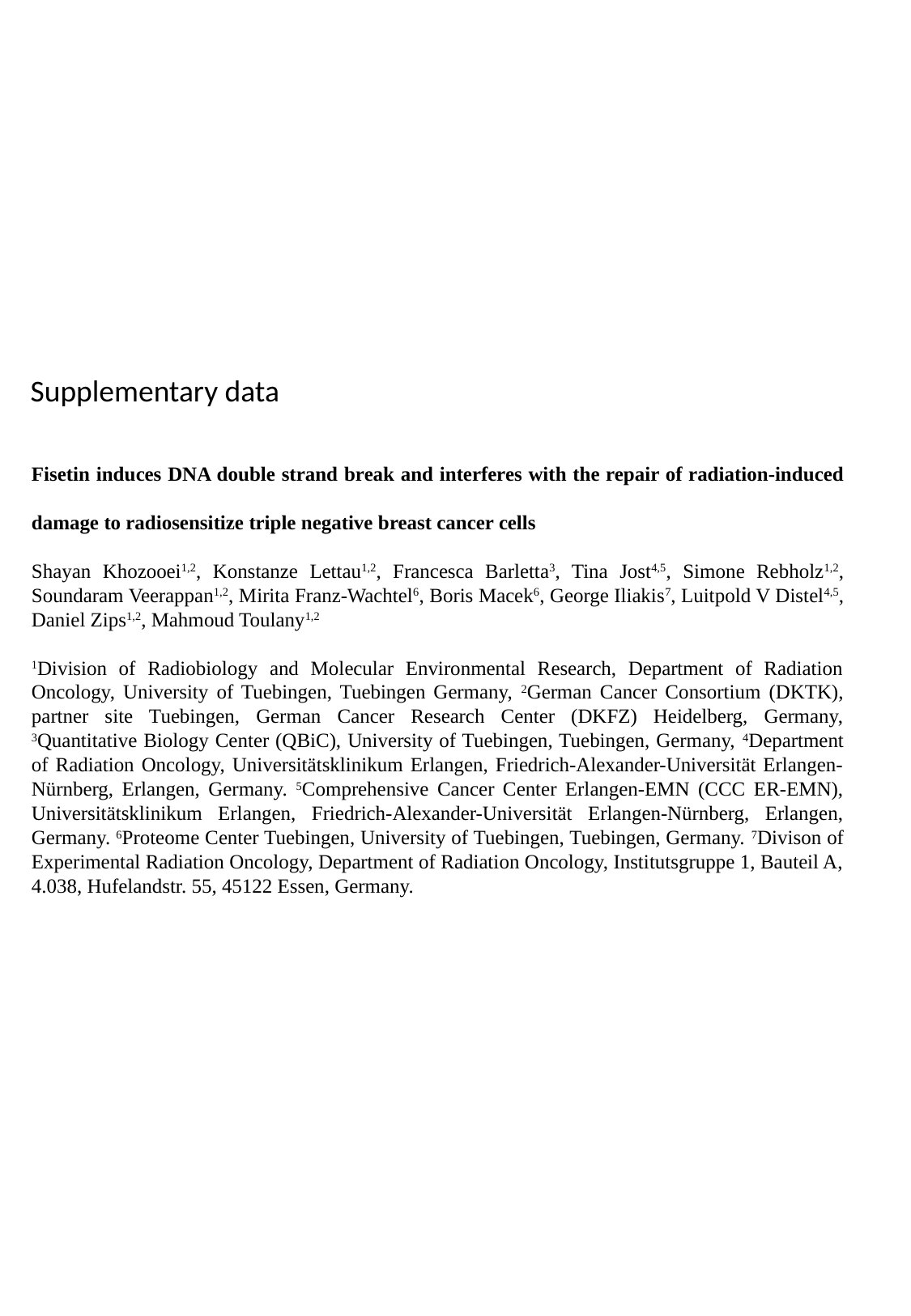

Supplementary data
­Fisetin induces DNA double strand break and interferes with the repair of radiation-induced damage to radiosensitize triple negative breast cancer cells
Shayan Khozooei1,2, Konstanze Lettau1,2, Francesca Barletta3, Tina Jost4,5, Simone Rebholz1,2, Soundaram Veerappan1,2, Mirita Franz-Wachtel6, Boris Macek6, George Iliakis7, Luitpold V Distel4,5, Daniel Zips1,2, Mahmoud Toulany1,2
1Division of Radiobiology and Molecular Environmental Research, Department of Radiation Oncology, University of Tuebingen, Tuebingen Germany, 2German Cancer Consortium (DKTK), partner site Tuebingen, German Cancer Research Center (DKFZ) Heidelberg, Germany, 3Quantitative Biology Center (QBiC), University of Tuebingen, Tuebingen, Germany, 4Department of Radiation Oncology, Universitätsklinikum Erlangen, Friedrich-Alexander-Universität Erlangen-Nürnberg, Erlangen, Germany. 5Comprehensive Cancer Center Erlangen-EMN (CCC ER-EMN), Universitätsklinikum Erlangen, Friedrich-Alexander-Universität Erlangen-Nürnberg, Erlangen, Germany. 6Proteome Center Tuebingen, University of Tuebingen, Tuebingen, Germany. 7Divison of Experimental Radiation Oncology, Department of Radiation Oncology, Institutsgruppe 1, Bauteil A, 4.038, Hufelandstr. 55, 45122 Essen, Germany.

## Slide 2
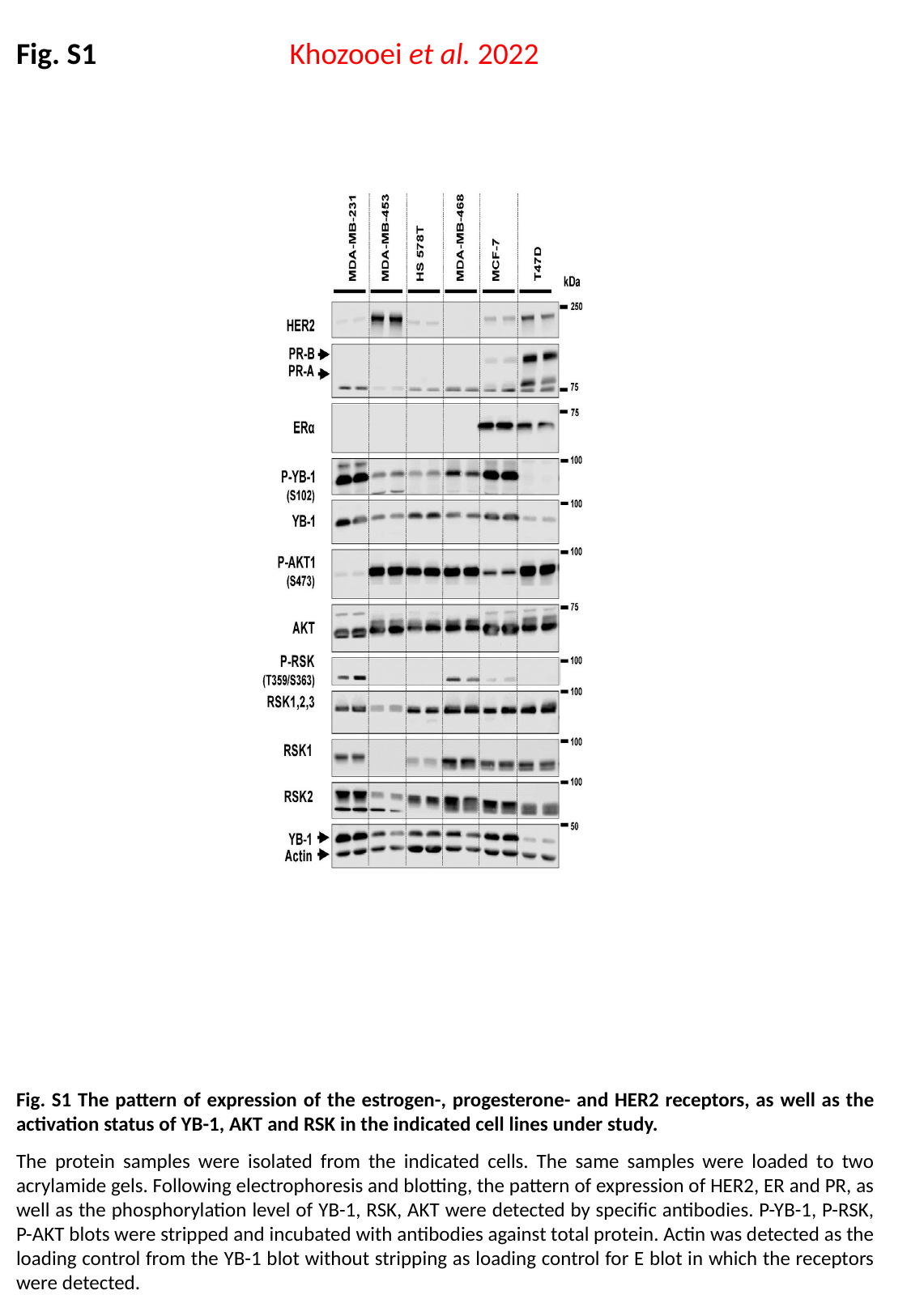

Fig. S1 Khozooei et al. 2022
Fig. S1 The pattern of expression of the estrogen-, progesterone- and HER2 receptors, as well as the activation status of YB-1, AKT and RSK in the indicated cell lines under study.
The protein samples were isolated from the indicated cells. The same samples were loaded to two acrylamide gels. Following electrophoresis and blotting, the pattern of expression of HER2, ER and PR, as well as the phosphorylation level of YB-1, RSK, AKT were detected by specific antibodies. P-YB-1, P-RSK, P-AKT blots were stripped and incubated with antibodies against total protein. Actin was detected as the loading control from the YB-1 blot without stripping as loading control for E blot in which the receptors were detected.

## Slide 3
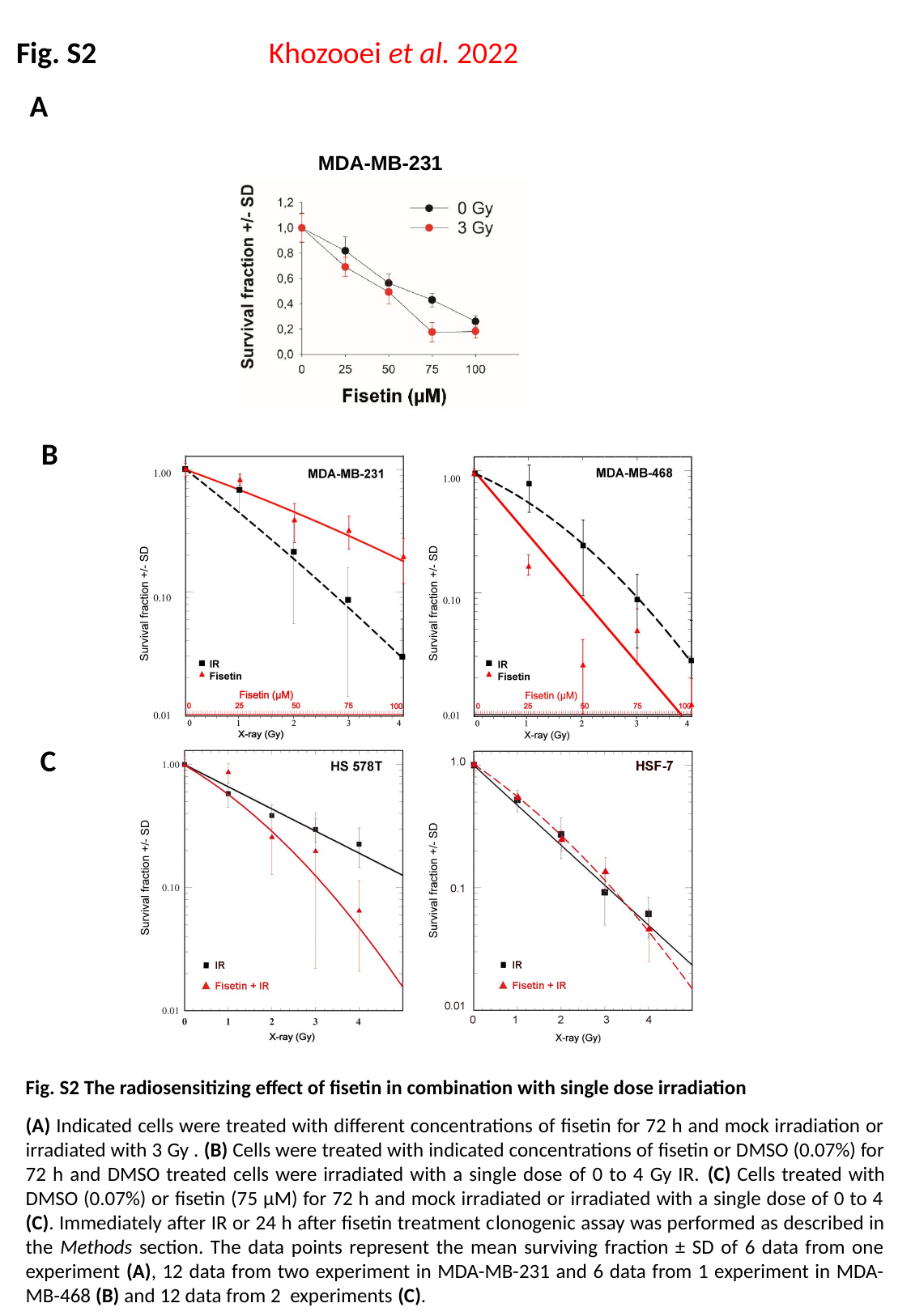

Fig. S2 Khozooei et al. 2022
A
MDA-MB-231
B
C
Fig. S2 The radiosensitizing effect of fisetin in combination with single dose irradiation
(A) Indicated cells were treated with different concentrations of fisetin for 72 h and mock irradiation or irradiated with 3 Gy . (B) Cells were treated with indicated concentrations of fisetin or DMSO (0.07%) for 72 h and DMSO treated cells were irradiated with a single dose of 0 to 4 Gy IR. (C) Cells treated with DMSO (0.07%) or fisetin (75 µM) for 72 h and mock irradiated or irradiated with a single dose of 0 to 4 (C). Immediately after IR or 24 h after fisetin treatment clonogenic assay was performed as described in the Methods section. The data points represent the mean surviving fraction ± SD of 6 data from one experiment (A), 12 data from two experiment in MDA-MB-231 and 6 data from 1 experiment in MDA-MB-468 (B) and 12 data from 2 experiments (C).

## Slide 4
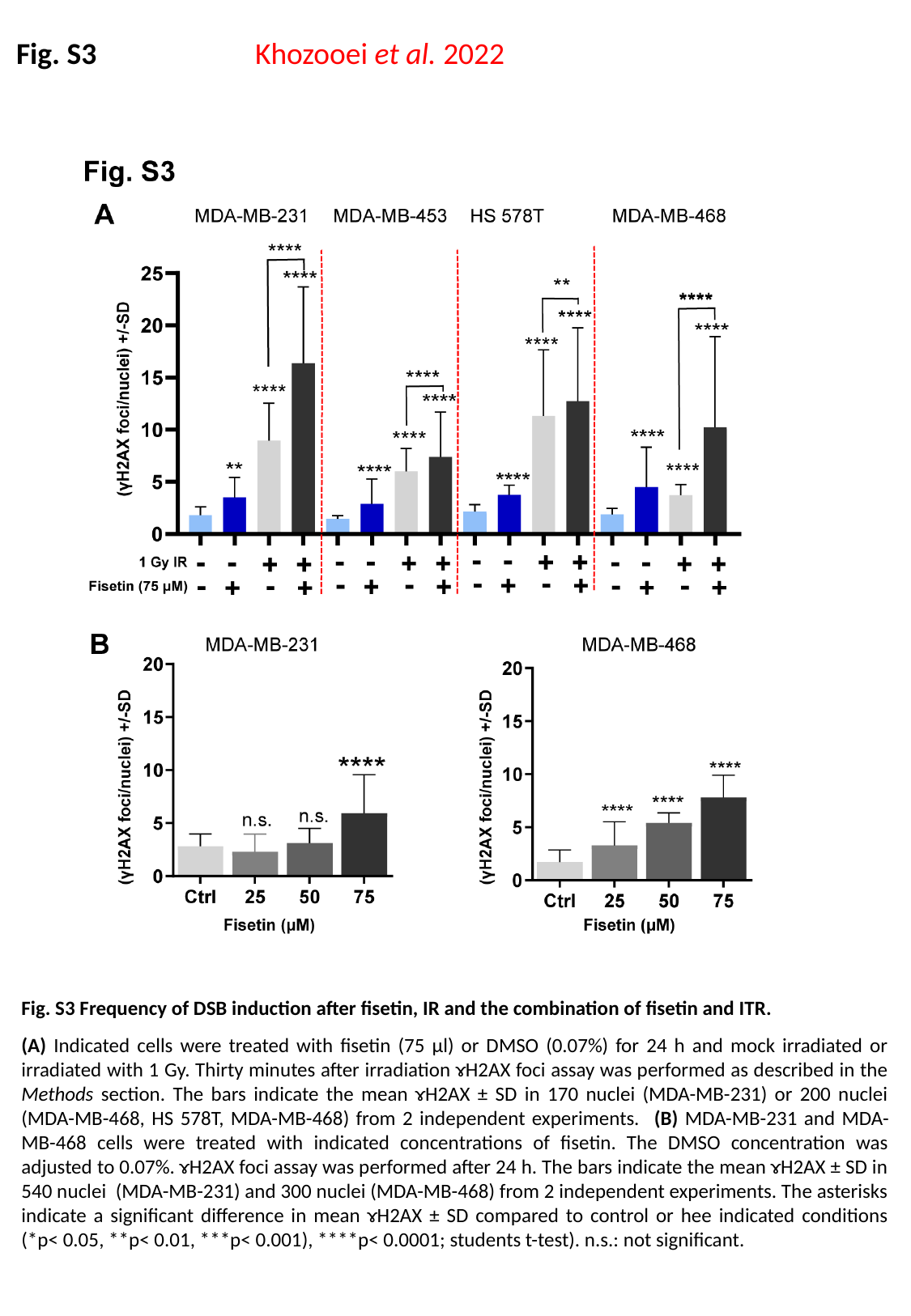

Fig. S3 Khozooei et al. 2022
Fig. S3 Frequency of DSB induction after fisetin, IR and the combination of fisetin and ITR.
(A) Indicated cells were treated with fisetin (75 µl) or DMSO (0.07%) for 24 h and mock irradiated or irradiated with 1 Gy. Thirty minutes after irradiation ɤH2AX foci assay was performed as described in the Methods section. The bars indicate the mean ɤH2AX ± SD in 170 nuclei (MDA-MB-231) or 200 nuclei (MDA-MB-468, HS 578T, MDA-MB-468) from 2 independent experiments. (B) MDA-MB-231 and MDA-MB-468 cells were treated with indicated concentrations of fisetin. The DMSO concentration was adjusted to 0.07%. ɤH2AX foci assay was performed after 24 h. The bars indicate the mean ɤH2AX ± SD in 540 nuclei (MDA-MB-231) and 300 nuclei (MDA-MB-468) from 2 independent experiments. The asterisks indicate a significant difference in mean ɤH2AX ± SD compared to control or hee indicated conditions (*p< 0.05, **p< 0.01, ***p< 0.001), ****p< 0.0001; students t-test). n.s.: not significant.

## Slide 5
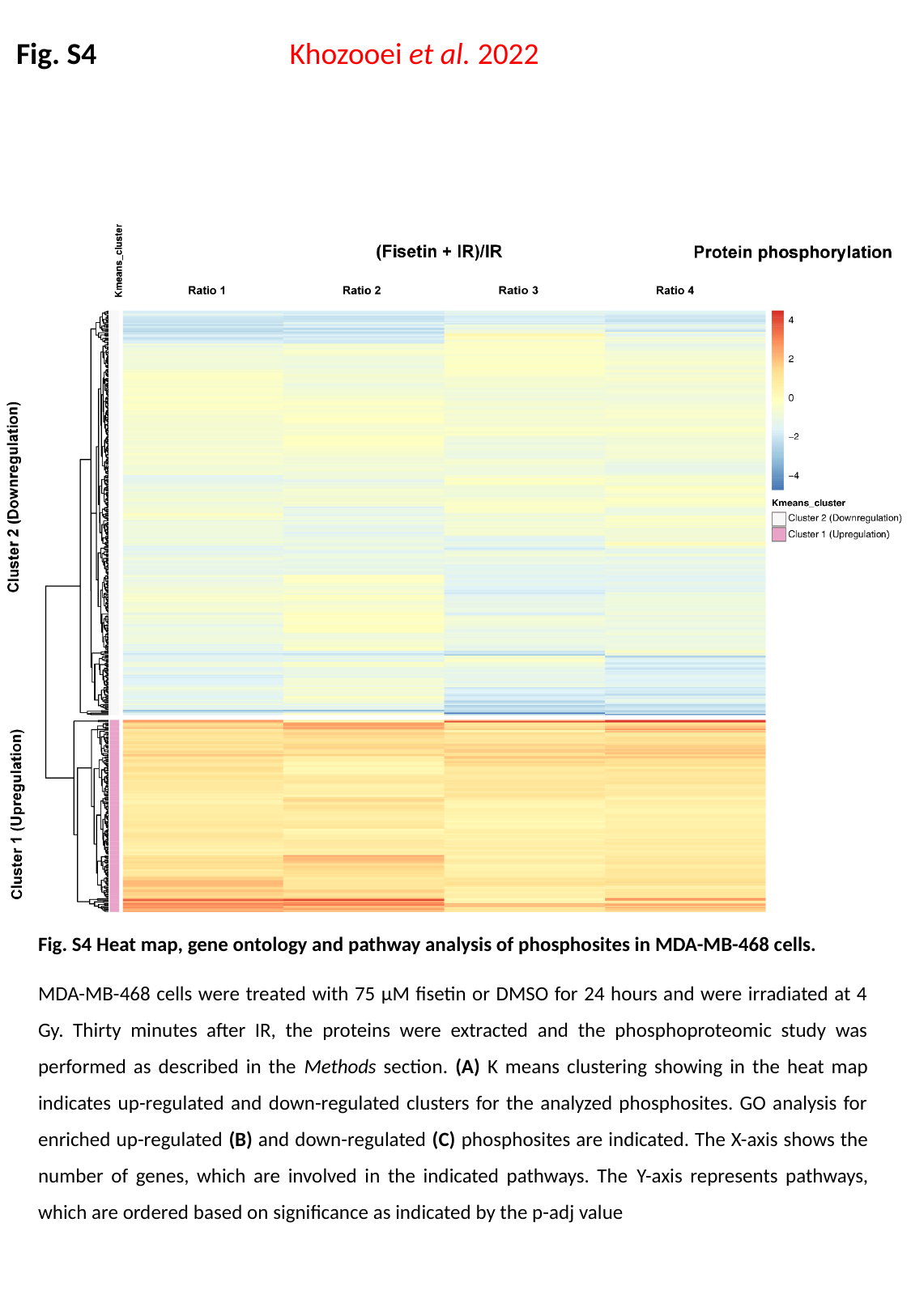

Fig. S4 Khozooei et al. 2022
Fig. S4 Heat map, gene ontology and pathway analysis of phosphosites in MDA-MB-468 cells.
MDA-MB-468 cells were treated with 75 µM fisetin or DMSO for 24 hours and were irradiated at 4 Gy. Thirty minutes after IR, the proteins were extracted and the phosphoproteomic study was performed as described in the Methods section. (A) K means clustering showing in the heat map indicates up-regulated and down-regulated clusters for the analyzed phosphosites. GO analysis for enriched up-regulated (B) and down-regulated (C) phosphosites are indicated. The X-axis shows the number of genes, which are involved in the indicated pathways. The Y-axis represents pathways, which are ordered based on significance as indicated by the p-adj value

## Slide 6
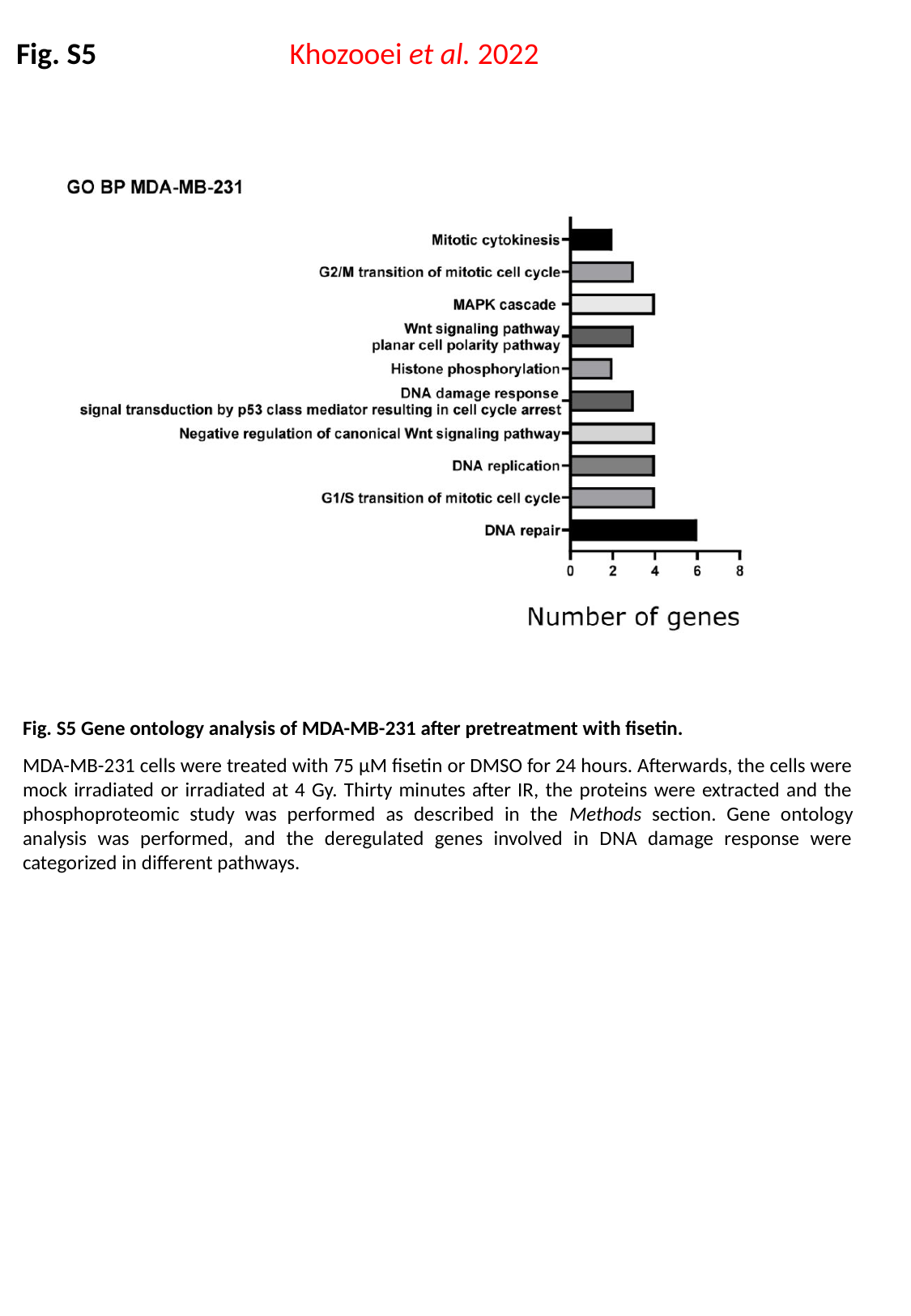

Fig. S5 Khozooei et al. 2022
Fig. S5 Gene ontology analysis of MDA-MB-231 after pretreatment with fisetin.
MDA-MB-231 cells were treated with 75 µM fisetin or DMSO for 24 hours. Afterwards, the cells were mock irradiated or irradiated at 4 Gy. Thirty minutes after IR, the proteins were extracted and the phosphoproteomic study was performed as described in the Methods section. Gene ontology analysis was performed, and the deregulated genes involved in DNA damage response were categorized in different pathways.
